# CLEC-2-dependent platelet subendothelial accumulation by flow disturbance contributes to atherogenesis in mice

**DOI:** 10.7150/thno.64601

**Published:** 2021-10-03

**Authors:** Chaojun Tang, Lei Wang, Yulan Sheng, Zhong Zheng, Zhanli Xie, Fan Wu, Tao You, Lijie Ren, Lijun Xia, Changgeng Ruan, Li Zhu

**Affiliations:** 1 Cyrus Tang Hematology Center, Cyrus Tang Medical Institute, Soochow University, Suzhou, China.; 2 Collaborative Innovation Center of Hematology of Jiangsu Province, Soochow University, Suzhou, China.; 3 Suzhou Key Lab for Thrombosis and Vascular Biology, Soochow University, Suzhou, China.; 4 Cardiovascular Biology Research Program, Oklahoma Medical Research Foundation, Oklahoma City, OK, USA.

**Keywords:** Atherosclerosis, CLEC-2, Disturbed flow, Platelets

## Abstract

**Rationale:** Platelets play an essential role in atherosclerosis, but the underlying mechanisms remain to be addressed. This study is to investigate the role of platelets in d-flow induced vascular inflammation and the underlying mechanism.

**Methods:** We established a disturbed blood flow (d-flow) model by partial carotid ligation (PCL) surgery using atherosclerosis-susceptible mice and wild-type mice to observe the d-flow induced platelet accumulation in the subendothelium or in the plaque by immunostaining or transmission electron microscopy. The mechanism of platelet subendothelial accumulation was further explored by specific gene knockout mice.

**Results:** We observed presence of platelets in atherosclerotic plaques either in the atheroprone area of aortic arch or in carotid artery with d-flow using *Ldlr^-/-^* or *ApoE^-/-^* mice on high fat diet. Immunostaining showed the subendothelial accumulation of circulating platelets by d-flow *in vivo*. Transmission electron microscopy demonstrated the accumulation of platelets associated with monocytes in the subendothelial spaces. The subendothelial accumulation of platelet-monocyte/macrophage aggregates reached peak values at 2 days after PCL. In examining the molecules that may mediate the platelet entry, we found that deletion of platelet C-type lectin-like receptor 2 (CLEC-2) reduced the subendothelial accumulation of platelets and monocytes/macrophages by d-flow, and ameliorated plaque formation in *Ldlr^-/-^* mice on high fat diet. Supportively, CLEC-2 deficient platelets diminished their promoting effect on the migration of mouse monocyte/macrophage cell line RAW264.7. Moreover, monocyte podoplanin (PDPN), the only ligand of CLEC-2, was upregulated by d-flow, and the myeloid-specific PDPN deletion mitigated the subendothelial accumulation of platelets and monocytes/macrophages.

**Conclusions:** Our results reveal a new CLEC-2-dependent platelet subendothelial accumulation in response to d-flow to regulate vascular inflammation.

## Introduction

Upon vascular injury, circulating platelets quickly adhere to injured sites to form hemostatic plug 1. Platelets also contribute to the inflammatory process that is critical in many pathological conditions including atherosclerosis 2, 3. Atherosclerosis preferentially develops at sites under disturbed blood flow (d-flow) which is nonlaminar flow with low speeds and chaotic directions 4-6. When exposed to d-flow, vascular endothelial cells undergo phenotypic changes, which leads to increased cytokine release and leukocyte recruitment 6, 7. Monocytes are the primary type of leukocytes that are transmigrated into inflamed vessel walls to become lipid-laden foam cells, a hallmark of atherosclerotic plaques 8, 9. Previous studies have revealed how d-flow activated endothelial cells lead to leukocyte recruitment during atherosclerosis 10, 11. However, how platelets respond to d-flow and thereby contribute to atherosclerosis remains unclear.

Previous *in vitro* studies showed that platelets are aggregated by collision in a region of d-flow 12 and the flow disturbance promotes adhesion of platelets on collagen to capture flowing leukocytes 13, 14. However, it is unknown whether these *in vitro* results recapitulate how platelets respond to d-flow *in vivo* where the shear stress and vasculature are quite different from those *in vitro* designs. Adhesion of platelets to the disturbed region was shown to promote formation of atheroma by facilitating monocyte adhesion 15. Platelet glycoprotein Ibα (GPIbα) and integrin αIIbβ3 are important in mediating platelet adhesion at vascular predilection sites in hyperlipidemic rabbits. Genetic deletion of endothelial von Willebrand factor (vWF), a ligand of platelet GPIbα, attenuates the development of atherosclerotic lesions at vascular sites exposed to d-flow in the aorta of *ApoE^-/-^* mice 16. A more recent study indicated that a platelet transcript signature was associated with macrophages in atherosclerotic plaques 17. However, how platelets contribute to the pathogenesis of atherosclerotic plaques *in vivo* remains to be addressed.

In this study, we determine how platelets contribute to vascular inflammation and atherosclerosis using a partial carotid ligation (PCL) induced d-flow model in the carotid artery in mice. We showed that flow disturbance induced platelet accumulation in the subendothelium and found that platelets were detected in the atherosclerotic plaques. Platelets associated with monocytes/macrophages in the subendothelium by d-flow, which was mediated by platelet C-type lectin-like receptor 2 (CLEC-2) and monocyte podoplanin (PDPN). Platelet CLEC-2 deficiency reduced plaque formation in atherosclerotic mice. Our results reveal a novel role for platelets to regulate vascular inflammation at sites where atherosclerosis is prone to develop.

## Results

### Platelets are detected inside atherosclerotic plaques

**Figure 1:**
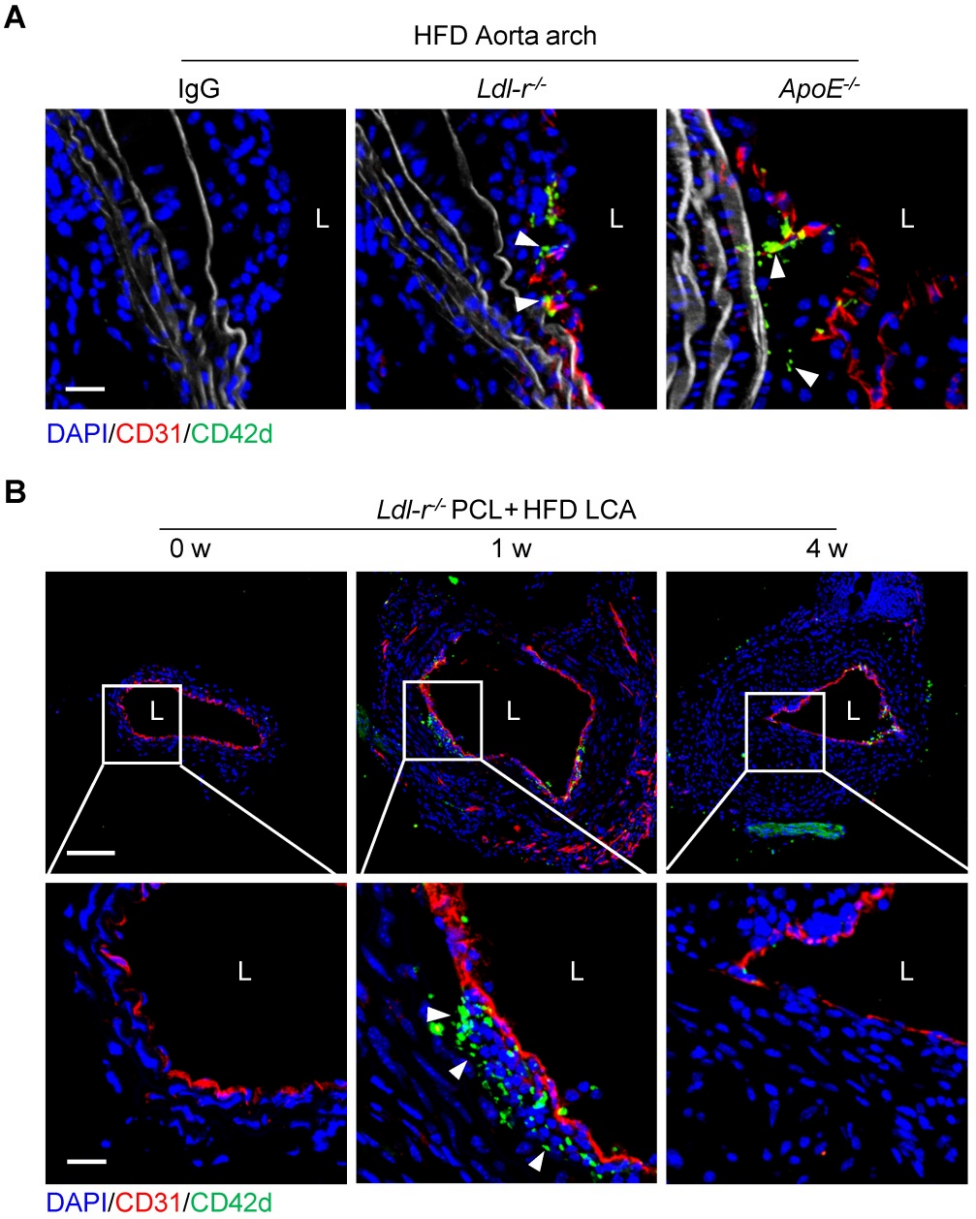
** Platelets are detected inside atherosclerotic plaques. (A)** The representative cross-sections of the aorta arch from *Ldl-r*^-/-^and *ApoE^-/-^* mice on high-fat diet (HFD) for 4 weeks stained for CD42d and CD31. The left panel shows an isotypic control for CD42d and CD31 antibodies on plaque stained for *Ldl-r*^-/-^ mice on HFD for 4 weeks. Bar = 20 μm. **(B)** The representative cross-sections of carotid plaques from *Ldl-r*^-/-^ mice on HFD for 1 and 4 weeks after partial carotid ligation (PCL) stained for CD42d and CD31. The bottom panel is the enlarged images of the boxed area in the upper panel. Bar = 100 μm (upper) or 20 μm (bottom). L, lumen. Green, CD42d; blue, DAPI; red, CD31; white, elastic fibers. White arrows indicate platelets.

Platelets have been known to play a role in atherogenesis. Atherosclerotic plaques usually develop at branches or curvature of arteries where disturbed blood flow (d-flow) occurs 18. To investigate the role of platelets in d-flow induced atherosclerosis, we examined the serial-sections of atherosclerotic plaques of the aortic arch of *Ldl-r^-/-^* or *ApoE^-/-^* mice fed high-fat diet (HFD) using immunostaining. Confocal imaging analysis revealed the presence of platelets in the plaques of the aortic arch isolated from either *Ldl-r^-/-^* or *ApoE^-/-^* mice after 4 weeks of HFD feeding (Figure 1A). To validate this, *Ldl-r^-/-^* mice were subjected to partial carotid ligation (PCL) to generate d-flow followed by 1 week or 4 weeks of HFD feeding to produce the carotid atherosclerosis (Figure S1). The ligated left common carotid artery (LCA) was isolated, and platelets were examined by immunofluorescence staining. Results showed that CD42d^+^ platelets were accumulated in the carotid plaques in mice with 1 week, but not 4 weeks, of HFD feeding after PCL (Figure 1B). Similarly, we observed CD42d^+^ platelets in carotid plaque in *ApoE^-/-^* mice with 1 week, but not 4 weeks, of HFD feeding after PCL (Figure S2A). We validated these results with antibodies to additional platelet markers such as CD41 and PF4. Immunostaining results of anti-CD41 or anti-PF4 showed the presence of platelets in atherosclerosis plaques (Figure S2B), which is consistent with our original findings. Our results provided evidence for a recent report that platelets associate with macrophages in the atherosclerotic plaques by single cell technology 17, and implied that platelets may present in the relatively early development of atherosclerosis.

### Flow disturbance induces platelet subendothelial accumulation in mice

**Figure 2:**
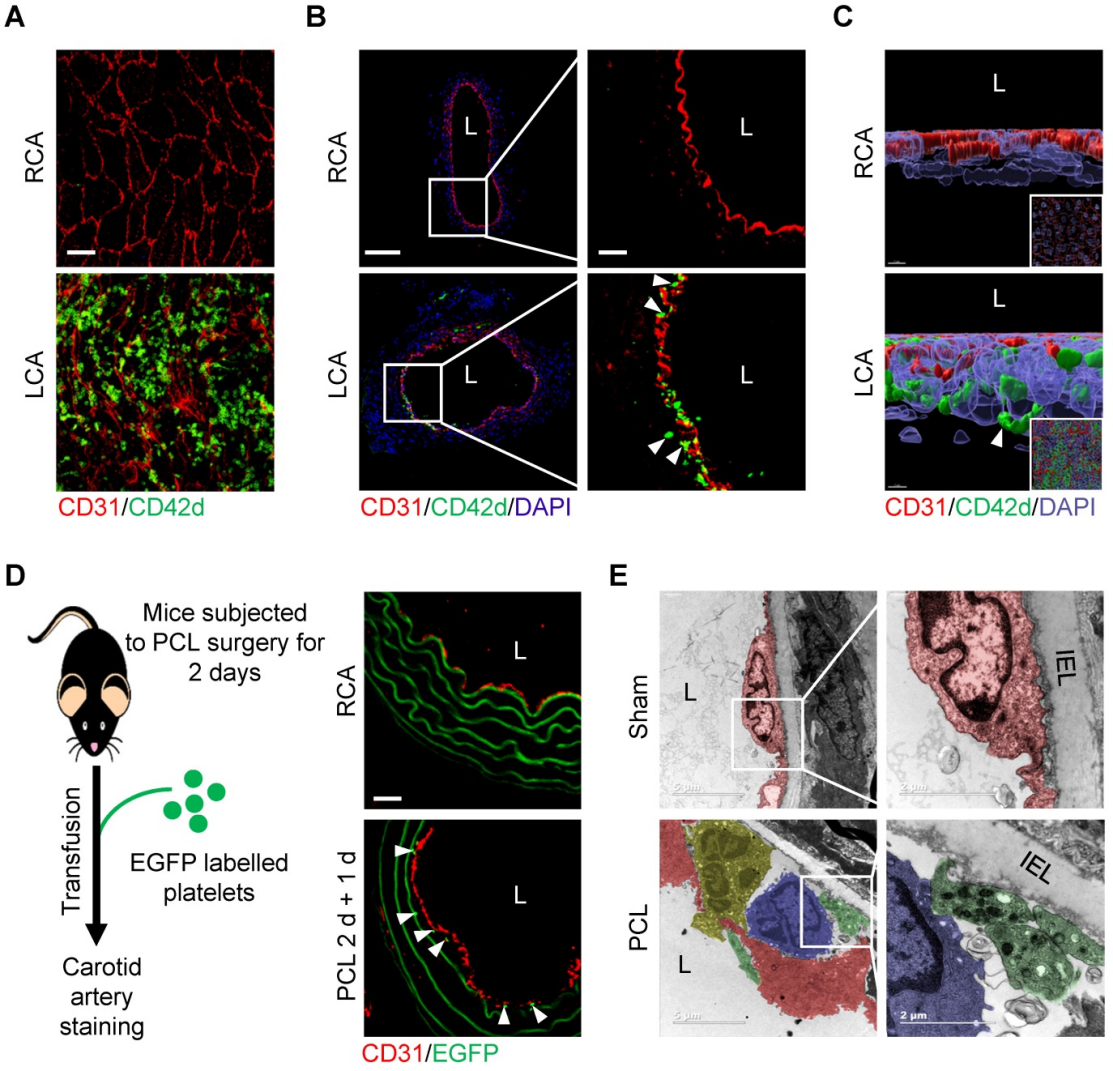
** Flow disturbance induces platelet subendothelial accumulation in mice. (A)** The representative *en face* images of the distal 1/3 of left common carotid artery (LCA, where d-flow usually occurs in PCL) and right common carotid artery (RCA) stained by CD31 and CD42d antibodies after PCL for 2 days. Bar = 20 μm. Green, CD42d; red, CD31. **(B)** The representative images of cross-section of LCA and RCA stained after PCL for 2 days. The enlarged images of the boxed area in the left panel were shown on the right. Bar = 100 μm (left) or 20 μm (right). Green, CD42d; red, CD31; blue, DAPI. L: lumen. White arrows indicate platelets in the subendothelium. **(C)** The representative 3D images of *en face* staining from RCA (upper) and LCA (bottom) after PCL for 2 days. The enlarged pictures are the original images in the bottom right corner that were rotated for 90 degrees. Bar = 7 μm. Green, CD42d; red, CD31; blue, DAPI. White arrows indicate platelets in the subendothelium. **(D)** EGFP^+^ platelets were infused into C57BL/6J mice 2 days after PCL and allowed to circulate for 24 hours. Left: schematic diagram of operation method. Right: representative images of cross-section staining from LCA with PCL or RCA. Red, CD31; green, EGFP^+^ platelets and elastic fibers. The white arrows point to EGFP^+^ platelets. **(E)** Representative TEM images of LCA upon PCL or sham control. The enlarged images of the boxed area in the left panel were shown on the right. Red, endothelial cells; green, platelets; yelllow, neutrophils; blue, monocytes. IEL, inner elastic layer. L, lumen. Bar = 5 μm (left) or 2 μm (right).

We then asked whether d-flow induces platelet accumulation in the subendothelial space using partial carotid ligation mouse model. Results showed that d-flow for 2 days induced a significant number of platelets in the subendothelial space in addition to adhering to the endothelium of LCA by whole-mount* en face* staining (Figure 2A) and immunofluorescence staining of tissue sections (Figure 2B). By contrast, platelets were not detected either on the endothelium or in the subendothelial space of right common carotid artery (RCA) control (Figure 2A, B). Further observation using three-dimensional (3D) reconstruction of confocal images confirmed the abundant CD42d^+^ platelet accumulation in the subendothelial space (Figure 2C, Figure S3 and Movie S1). To validate the subendothelial accumulation of circulating platelets, we infused EGFP-expressing platelets into the mice that have been subjected to PCL for 2 days. One day after transfusion, we examined whether EGFP-labeled platelets were accumulated under the endothelium in the d-flow affected region of the carotid artery (Figure 2D, left). Results showed that EGFP-expressing platelets were detected under the endothelium of LCA, but not RCA, after PCL (Figure 2D, right). Furthermore, observation of the ultrastructure of cells in the vasculature of the d-flow region by transmission electron microscopy (TEM) showed that d-flow induced the deformation of endothelium and the accumulation of leukocytes and platelets in the enlarged subendothelial spaces. Notably, platelets were potentially in contact with monocytes (Figure 2E).

### Platelets in the subendothelial space are associated with monocytes/macrophages

**Figure 3:**
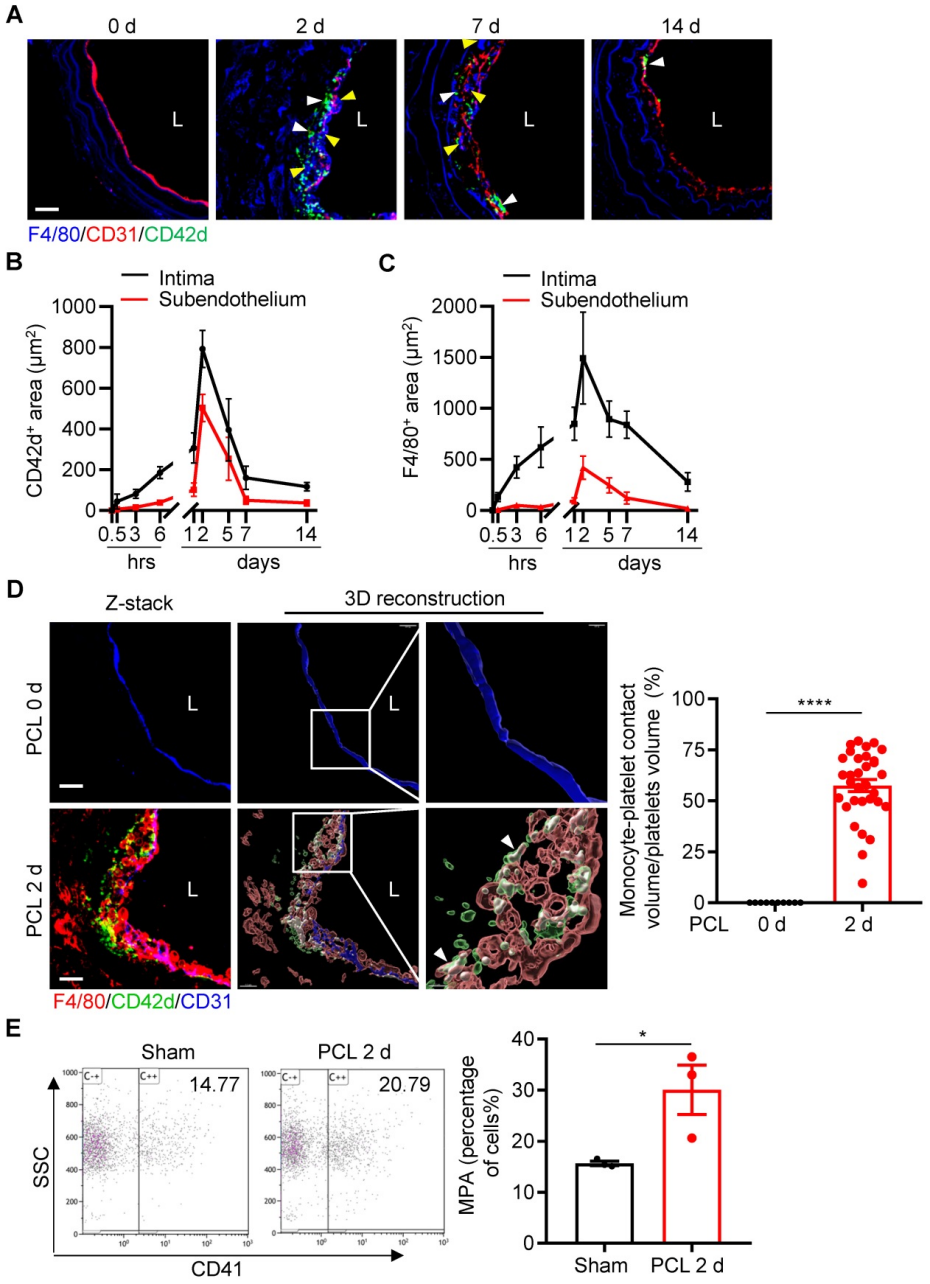
**Platelet subendothelial accumulation by d-flow associates with monocytes/macrophages. (A)** The representative images of cross-section staining from LCA of PCL at different time points (0 h, 2 d, 7 d, 14 d) after PCL for monocyte and platelet accumulation. L, lumen. Green, CD42d; red, CD31; blue, F4/80 and elastic fibers. White arrows indicate platelets; yellow arrows indicate monocytes/macrophages. **(B, C)** The chart represents the infiltration of platelets (B) or monocytes (C) in intima (black line) and subendothelium (red line) at different time points after PCL (the images for 0.5 h, 3 h, 6 h, 1 d, and 5 d are shown in the Figure S4). The ordinates represent the area of infiltrated cells and the abscissae represent the time of PCL surgery. N ≥ 4 mice per group. **(D)** The representative images of cross-section staining and 3D reconstruction image from LCA after PCL for 0 or 2 days to observe platelet-monocyte aggregates. The enlarged images of the boxed area in the middle panel were shown on the right. L, lumen. Green, CD42d; red, F4/80; blue, CD31; white arrows point to the contact area of platelets-monocytes. Bar = 20 μm. Right: the quantitative statistics of the contact volume ratio of platelets with monocytes in blood vessels after PCL for 0 or 2 days by Imaris 9.5. N = 32 fields from 5 mice per group.** (E)** Monocyte-platelet aggregates (MPA) from peripheral blood of mice with or without PCL for 2 days were analyzed by flow cytometry and shown as the percentage of total monocytes. N = 3 mice per group. Data are mean ± SEM. *P < 0.05, ****P < 0.0001, by unpaired t-test.

Platelets are known to interact with leukocytes and facilitate leukocyte recruitment during inflammation 2. We next examined the dynamic changes of platelet subendothelial accumulation and their association with leukocyte infiltration under d-flow. By confocal microscopic analysis of immunostained cross-sections of carotid arteries at different time points after PCL, we found that the maximal infiltrations for platelets and monocytes occurred at 2 days post PCL (Figure 3A and Figure S4). Quantitative analysis showed that the intima and subendothelial accumulation of platelets and F4/80^+^ monocytes/macrophages emerged at 6 hours, reached a peak at 2 days, and maintained a certain level at 7 days post PCL (Figure 3B, C). In addition, we also observed the infiltration of neutrophils in the vascular intima after PCL surgery. Neutrophils began to adhere to the damaged blood vessel wall relatively early (at 30 minutes post PCL) and stopped accumulating in the subendothelium (back to baseline) at 7 days after PCL (Figure S5A, B), indicating a predominant early transmigration of neutrophils in d-flow stimulation. Further examination by 3D reconstruction showed that the accumulated platelets were in contact with monocytes/macrophages (F4/80^+^) or neutrophils (Ly6G^+^), forming monocyte/macrophage-platelet or neutrophil-platelet aggregates after PCL as analyzed by Imaris 9.5 (Figure 3D left and Figure S5C). The percent of platelets aggregated with monocytes/macrophages over total platelets in the affected region at 2 days after PCL (57.48%, Figure 3D right) was significantly higher than that of neutrophils (26.17%) (Figure S5D and Movie S2), implying that platelets mainly interact with monocytes/macrophages during platelet subendothelial accumulation. Additionally, we examined whether d-flow induced an increase in circulating monocyte-platelet aggregates (MPA) as circulating MPA is a marker of the pathogenesis of cardiovascular diseases such as atherosclerosis 19, 20. Blood was collected from mice before and at 2 days post PCL, and MPA were detected by flow cytometry. Ly6C and CD11b double-positive cells were defined as monocytes. Compared with the control group without PCL surgery, the ratio of MPA to total monocytes in circulating blood was increased by 47.76% (P = 0.0413) 2 days after PCL (Figure 3E). Together, these results support the notion that platelet subendothelial accumulation associates with monocytes/macrophages under d-flow.

### Loss of platelet CLEC-2 decreases platelet and monocyte/macrophage interaction and their subendothelial accumulation

**Figure 4:**
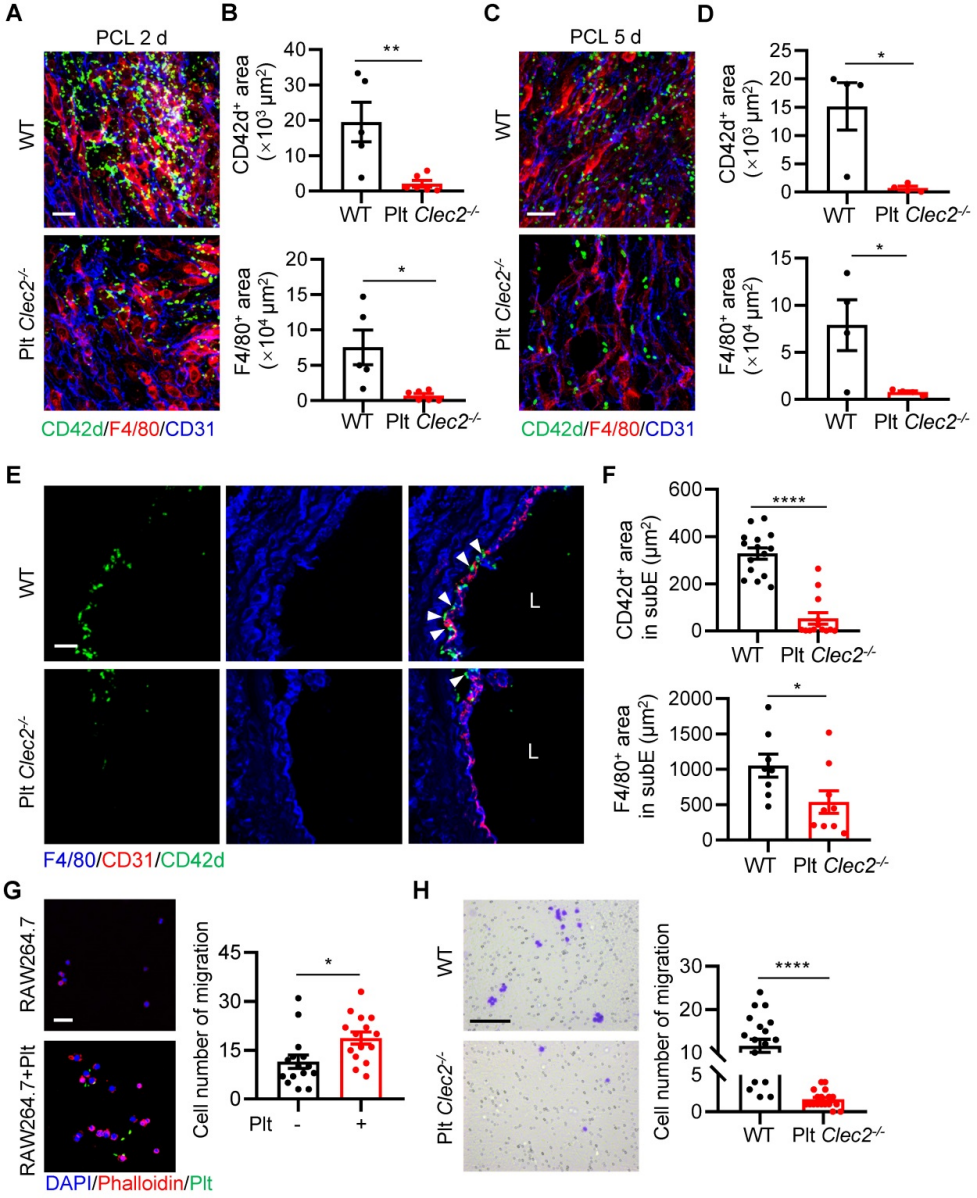
** Loss of platelet CLEC-2 reduces monocyte/macrophage and platelet interaction and their subendothelial accumulation. (A)** The representative *en face* staining images of the infiltrated region of LCA after PCL 2 days from WT or Plt* Clec2^-/-^* mice. Green, CD42d; red, F4/80; blue, CD31. Bar = 20 µm. **(B)** The quantitative analysis of CD42d^+^ platelet area (upper) and F4/80^+^ monocyte/macrophage area (bottom) in the distal of LCA (where d-flow usually occurs in PCL) from WT or Plt *Clec2^-/-^* mice 2 days after PCL. N ≥ 5 mice per group. Representative *en face* images of LCA are shown in Figure S6C. **(C, D)** The representative *en face* staining images of the infiltrated region of LCA (C) and quantitative analysis of CD42d^+^ platelet area (upper) and F4/80^+^ monocyte/macrophage area (bottom) of LCA (D) after PCL 5 days from WT or Plt* Clec2^-/-^* mice. N = 4 mice per group. Representative *en face* images of LCA are shown in Figure S6D. Green, CD42d; red, F4/80; blue, CD31. Bar = 20 µm. **(E)** The representative images of section staining of LCA from WT or Plt* Clec2^-/-^* mice 2 days after PCL. L, lumen. Green, CD42d; blue, F4/80; red, CD31. Bar = 20 μm. White arrows indicate platelets. **(F)** The quantitation analysis of subendothelial platelets (upper) and monocytes/macrophages (bottom) fluorescence area. N ≥ 8 fields from 3 mice per group. subE, subendothelium. **(G)** Left: the representative images of RAW264.7 cell migration affected by platelet incubation in transwell migration assay. Right: quantitative analysis of migrated RAW264.7 cells in different groups. Bar = 100 μm. Blue, DAPI; green, platelet; red, phalloidin. Plt: platelet; N = 15 fields per group.** (H)** Left: the representative images of migrated RAW264.7 cells affected by incubation with *Clec2^-/-^* platelets in transwell migration assay. Right: quantitative analysis of migrated RAW264.7 cells in different groups. Bar = 100 μm. N ≥ 18 fields per group. Data are mean ± SEM. *P < 0.05, **P < 0.01, ****P < 0.0001, by unpaired t-test.

We next asked what type of molecules mediates the platelet-monocyte interaction and subendothelial accumulation induced by d-flow. Platelet CLEC-2 has been found to regulate vascular integrity 21, 22. However, whether CLEC-2 contributes to d-flow induced platelet and monocyte interaction and their subendothelial accumulation is unknown. To test whether platelet CLEC-2 regulates platelet subendothelial accumulation induced by d-flow *in vivo*, we used our previously established mouse model (*Clec2^fl/fl^*; *Pf4-Cre*) in which platelet CLEC-2 was specifically deleted by breeding *Clec2^fl/fl^* mice with PF4-Cre transgenic mice 23. In addition, we confirmed CLEC-2 depletion in platelets by flow cytometry (Figure S6A, B). The *en face* staining image of the LCA infiltration area after PCL showed that platelets accumulated in the form of either MPA or alone in the region of deformed endothelium of WT mice, while a lack of CLEC-2 on platelets significantly inhibited platelet and monocyte accumulation on endothelial cells (Figure 4A). Statistical analysis on the infiltration area showed that accumulation of CD42d^+^ platelets and F4/80^+^ monocytes/macrophages in LCA of Plt *Clec2^-/-^* mice was reduced by 89.40% (P = 0.0080) and 90.60% (P = 0.0137), respectively, at 2 days after PCL compared to those of WT mice (Figure S6C and Figure 4B). Similarly, when the disturbed flow lasted for 5 days, the accumulation of CD42d^+^ platelets and F4/80^+^ monocytes/macrophages in LCA of Plt* Clec2^-/-^* mice was reduced by 95.35% (P = 0.0135) and 90.20% (P = 0.0393), respectively, compared to those of WT mice, although the number of infiltrated platelets and monocytes were both significantly reduced, compared to PCL for 2 days (Figure S6D and Figure 4C, D). In LCA cross-sections, the accumulation of platelets and monocytes/macrophages in the subendothelium was reduced by 83.77% and 49.06%, respectively, in Plt *Clec2^-/-^* mice compared to those of WT mice 2 days after PCL (Figure 4E, F). To examine whether CLEC-2 contributes to d-flow induced platelet and monocyte interaction, we used mouse monocyte/macrophage cell line RAW264.7 and found that platelet incubation promoted the migration of RAW264.7 (P = 0.0137, Figure 4G), while incubation with CLEC-2 deficient platelets reduced RAW264.7 cell migration (P < 0.0001, Figure 4H). Together, our results indicate that CLEC-2 plays a major role in mediating monocyte-platelet interaction and their subendothelial accumulation by d-flow.

### Podoplanin deletion mitigates d-flow-induced subendothelial accumulation of platelets and monocytes/macrophages

**Figure 5:**
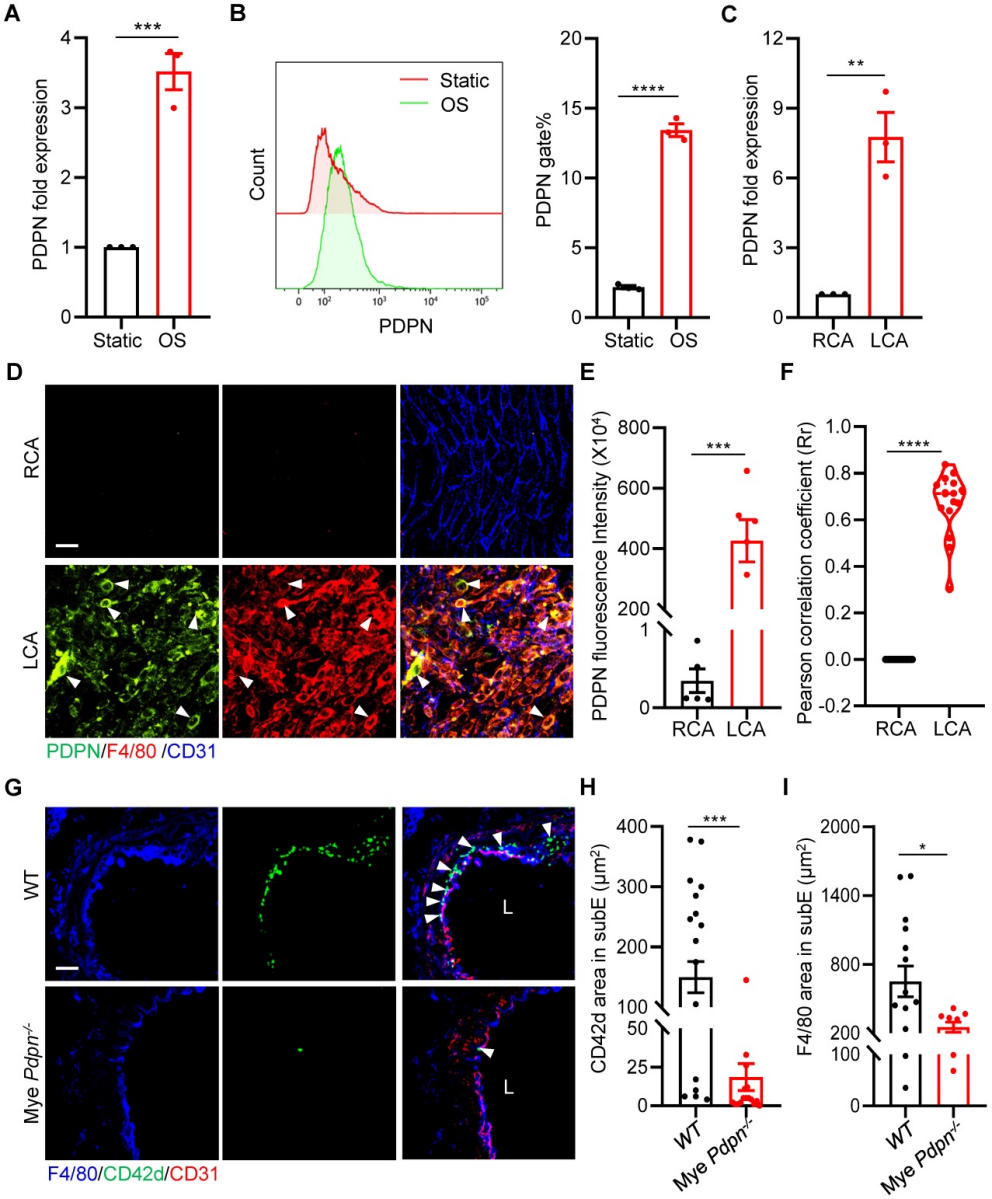
** Podoplanin deletion mitigates d-flow-induced subendothelial accumulation of platelets and monocytes/macrophages. (A, B)** RAW264.7 cells were treated for 12 h by oscillatory shear (OS) following laminar shear (LS) treatment for 12 h. The expression of PDPN were examined by qRT-PCR (A) and flow cytometry (B). In (B), a representative half-shift histogram was shown in the left. N = 3 per group. Results are representative of 3 independent experiments. **(C)** The relative RNA expression level of PDPN in LCA treated with PCL for 2 days was evaluated using real-time RT-PCR with RCA as control. Each sample was collected from 4-5 mice. **(D)** Representative images of *en face* staining of PDPN expression from LCA after PCL for 2 days. Green, PDPN; red, F4/80; blue, CD31. Bar = 20 μm. White arrows indicate monocyte expressing PDPN. **(E)** Quantitative statistical analysis of PDPN fluorescence intensity. N ≥ 5 fields per group. **(F)** Co-localization analysis of monocytes/macrophages and PDPN by cellSens 3.1. The ordinate represents Pearson correlation coefficient (PCC; Rr), the abscissa represents the group. N = 16 fields from 3 mice per group. **(G)** The representative images of cross-section staining of LCA for platelet and monocyte/macrophage accumulation from Mye *Pdpn^-/-^* and WT littermate mice after PCL for 2 days. L, lumen. Green, CD42d; blue, F4/80; red, CD31. Bar = 20 μm. White arrows indicate platelets. **(H, I)** Quantitative analysis of CD42d^+^ (H) and F4/80^+^ (I) fluorescence area in the subendothelium. N ≥ 9 fields from 3 mice per group. subE: subendothelium. In all relevant panels, data are mean ± SEM. *P < 0.05, **P < 0.01, ***P < 0.001, ****P < 0.0001, by unpaired t-test.

To investigate how platelet CLEC-2 mediates monocyte-platelet aggregate formation, we examined whether d-flow induces monocyte expression of podoplanin (PDPN), a small mucin-type transmembrane glycoprotein and a known CLEC-2 ligand, which was reported to express on monocytes/macrophages during inflammation 24, 25. We first analyzed the expression of PDPN of RAW264.7 cells after 12 hours of stimulation with oscillating flow (OS). The results of qPCR showed that the expression of PDPN in RAW264.7 cells was up-regulated by approximately 3-fold (P = 0.0009) after OS stimulation (Figure 5A), compared with static cultured cells. Similarly, the protein level was significantly increased by approximately 6-fold based on flow cytometry analysis (P < 0.0001, Figure 5B). Importantly, the increased expression of PDPN was validated in primary mouse peritoneal macrophages after OS stimulation (Figure S7A, B). We then examined the vascular expression of podoplanin using qPCR and immunostaining in vascular intima and media after PCL *in vivo*. The results of qPCR showed that after 2 days of d-flow stimulation, the expression of vascular podoplanin was up-regulated by approximately 6-fold (P = 0.0031) compared with the control RCA (Figure 5C). *En face* staining also showed that d-flow induced vascular expression of PDPN (Figure 5D, E). Further analysis by cellSens 3.1 showed a higher PCC value at 2 days after PCL compared to the non-PCL group (P < 0.0001), indicating that PDPN is highly expressed in F4/80^+^ monocytes/macrophages stimulated by d-flow (Figure 5D, F).

To examine whether monocyte/macrophage podoplanin mediates platelet and monocyte/macrophage subendothelial accumulation by d-flow, we used an established mouse model (*Pdpn^fl/fl^*; *LysM-cre*) that we published previously, in which myeloid podoplanin was specifically deleted by breeding *Pdpn^fl/fl^* mice with LysM-Cre transgenic mice, of which podoplanin is deleted in monocytes, neutrophils, and mature macrophages^23^. Compared with *Pdpn^fl/fl^* mice (WT), *Pdpn^fl/fl^*; *LysM-cre* mice (Mye* Pdpn^-/-^*) had significantly reduced PDPN protein levels in monocyte/macrophages (Figure S7C). In LCA cross-sections at 2 days after PCL, intimal accumulation of platelets and monocytes/macrophages was reduced by 87.59% (P = 0.0002) and 61.36% (P = 0.0353), respectively, in Mye *Pdpn^-/-^* mice compared to WT littermates (Figure 5G-I), indicating that monocyte/macrophage podoplanin mediates subendothelial platelet and monocyte/macrophage accumulation by d-flow *in vivo*.

### Platelet CLEC-2 deficiency attenuates d-flow induced atherosclerotic plaque formation

**Figure 6:**
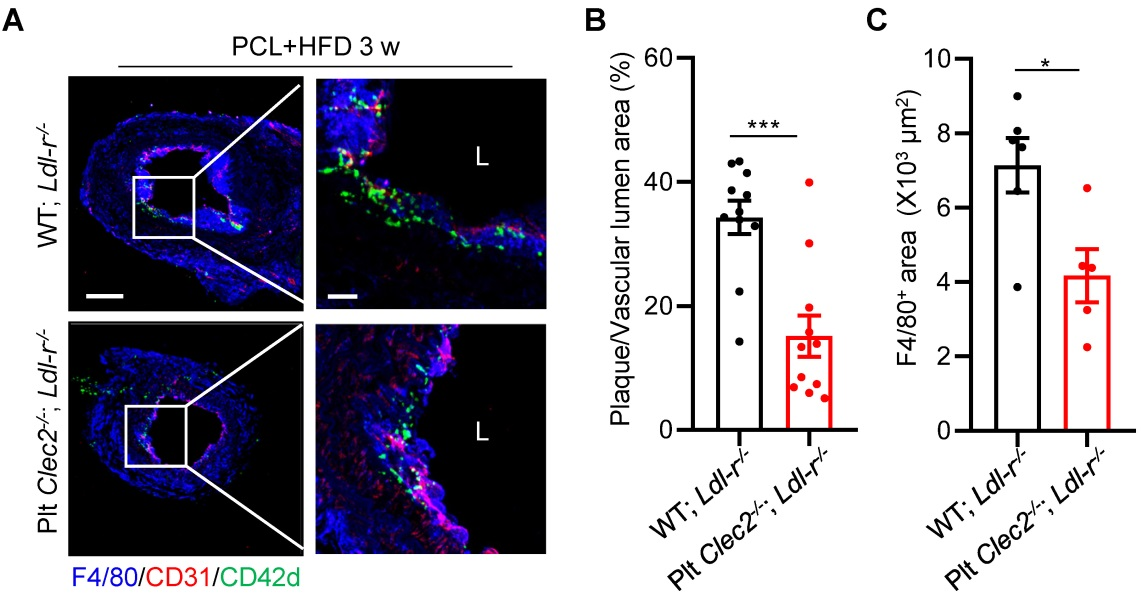
** Platelet CLEC-2 deficiency attenuates d-flow induced atherosclerotic plaque formation.** Platelet CLEC-2 deficient chimeric *Ldl-r^-/-^* mice were generated by transplanting bone marrow cells from WT and Plt* Clec2^-/-^* mice into irradiated *Ldl-r^-/-^* mice.** (A)** The representative IFC images of common carotid artery plaque areas in WT; *Ldl-r^-/-^* or Plt* Clec2^-/-^*;* Ldl-r^-/-^* mice on HFD for 3 weeks after PCL. Red bar = 100 μm; white bar = 20 μm. L, lumen. Green, CD42d; red, CD31; blue, F4/80. The right panel is the enlarged image of the boxed area in the left panel. Red bar = 100 μm, white bar = 20 μm. **(B)** The ratio of plaque area versus vascular lumen area was quantitatively analyzed. N = 11 fields from 3 mice per group.** (C)** Quantitation analysis of macrophage infiltrated area. N ≥ 5 fields from 3 mice per group. Data are mean ± SEM. *P < 0.05, **P < 0.01, by unpaired t-test.

Finally, we determined if lack of platelet CLEC-2 reduces atherogenesis. Platelet CLEC-2-deficient chimeric *Ldlr^-/-^* mice were generated by bone marrow transplantation from Plt* Clec2^-/-^* mice into *Ldlr^-/-^* mice. Bone marrow transplantation was successful as demonstrated by less than 1% expression of CLEC-2 of peripheral platelets of the recipient mice after bone marrow reconstitution (Figure S8). Mice were subjected to PCL and fed with HFD for 3 weeks. Results showed that the ratio of plaque area versus vascular lumen area and the accumulation of macrophages were reduced by 55.81% (P < 0.001, Figure 6A, B) and by 41.59% (P = 0.0187, Figure 6A, C), respectively, in Plt* Clec2^-/-^* transplanted *Ldlr^-/-^* mice (Plt *Clec2^-/-^*; *Ldlr^-/-^*) compared to WT transplanted* Ldlr^-/-^* mice (WT;* Ldlr^-/-^*). These results indicate that platelet CLEC-2 deficiency reduces atherosclerotic plaque formation by d-flow, likely by inhibiting platelet and monocyte/macrophage subendothelial accumulation.

## Discussion

Flow disturbance occurs in the branched points and curvatures of large arteries, which is prone to atherosclerosis. In this study, we detected platelets in the atheroprone area of aortic arch in *ApoE^-/-^* or *Ldl-r^-/-^* mice and in the plaques of carotid artery of PCL model on high fat diet. Furthermore, we uncovered that d-flow induces platelet accumulation in the subendothelial space. Interaction of platelet CLEC-2 with monocyte/macrophage PDPN mediated platelet and monocyte/macrophage accumulation. Platelet-monocyte interaction facilitated monocyte/macrophage transmigration in a CLEC-2-dependent manner. To our knowledge, this is the first observation that platelets accumulate in the subendothelium of large arteries under blood flow disturbance, a potential mechanism for platelets to contribute to d-flow-induced atherosclerosis.

We observed subendothelial accumulation of monocyte/macrophage and platelet aggregates, and individual platelets by d-flow stimulation. The formation of leukocyte-platelet aggregates is involved in a variety of inflammatory processes 26, and the formation of monocyte-platelet aggregates is currently considered to be one of the primary features of cardiovascular disease such as atherosclerosis 17, 19. As leukocytes move via diapedesis through tightly opposed endothelial borders 27, platelet-leukocyte aggregates presumably cross the endothelium via a similar mechanism. Recently, an article reported that laminar blood flow protects the endothelial barrier function and maintains levels of tight junctions ZO-1 and occludin proteins 28. We also observed that the flow restriction achieved by partial ligation of the carotid artery caused defects in the endothelial lining, which is manifested as a discontinuity in the PECAM staining (Figure 3A). Therefore, we speculate that individual platelets may be “flushed” through the gaps in the injured endothelial monolayer passively. On the other hand, platelets may actively transmigrate into the subendothelial space. Some recent studies have shown that platelets are capable of migrating toward bacteria or chemokines either *in vivo* or* in vitro* 29-31. In this study, using a mouse model to integrate surgically-induced d-flow in carotid artery with 3D image reconstruction and TEM, we provided novel evidence of platelet subendothelial accumulation in the d-flow-induced vascular inflammation *in vivo*. To support evidence that d-flow induces subendothelial platelet accumulation, we examined whether the LPS-induced vascular inflammation play a similar role as d-flow. LPS injection was confirmed by plasma levels of inflammation factors, such as TNF-α, IL-1β. Results showed that LPS did not induce platelet subendothelial accumulation (Figure S9), indicating that platelet subendothelial accumulation by d-flow is selective. However, how individual platelets selectively transmigrate through endothelium in response to d-flow and accumulate in the subendothelium remains to be investigated. We are in the process of developing protocols for real-time microscopic imaging of platelet transmigration in the carotid artery *in vivo* or *ex vivo* after d-flow to address this important question.

Platelets participate in the development of atherosclerosis by mediating interactions between platelets and leukocytes or between platelets and endothelial cells 32, 33. Of note, P-selectin on activated platelets or endothelial cells binds PSGL-1 on leukocytes especially neutrophils and monocytes, thereby promoting the leukocyte adhesion and transmigration during inflammation. In our study, 2 days after PCL is the peak time for platelets and leukocytes, especially monocytes, to form aggregates and accumulate under the intima. We found that lack of PSGL-1 had no significant effect on the accumulation of platelet-monocytes 2 days after PCL (Figure S10). These results suggest that there are other unknown mechanisms involved in d-flow-induced platelet-monocyte interaction.

PDPN, the only endogenous ligand of CLEC-2, is expressed on many cell types including monocytes/macrophages during inflammation 25. CLEC-2/PDPN interaction plays an important role in lymphangiogenesis, vascular integrity, and immune response 21, 34, 35. Our results in this study indicate that interaction of platelet CLEC-2 with PDPN on monocytes/macrophages plays a major role in the formation of monocyte/macrophage-platelet aggregates and their subendothelial accumulation in the development of atherosclerosis in mice. It should be noted that PDPN is expressed on fibroblastic reticular cells in the lymph node 36 and stromal cells in the blood vessel, which extends processes into the endothelial layer, potentially interacting with circulating platelets via CLEC-2 37. Controversially, a recent single-cell analysis indicated the absence of typical mesenchymal stromal cells or fibroblastic reticular cells on the inner layer of the aorta arteries in mice 38. Our data demonstrate that PDPN is not expressed on normal blood vascular endothelial cells nor on the inflamed endothelial cells induced by d-flow (data not shown).

Although the role of platelets in atherosclerosis has long been recognized, the detailed mechanism has not been fully elucidated. Barrett et al. recently reported that mice with high cholesterol have increased macrophage-platelet aggregates in atherosclerotic plaques and that platelets promote monocyte recruitment to plaques 17. Consistent with this, we showed that d-flow induces monocyte/macrophage-platelet aggregate formation in the circulation or subendothelium and the presence of platelets in atherosclerotic plaques. Moreover, the interaction of platelet CLEC-2 with monocyte/macrophage PDPN mediates d-flow-induced monocyte/macrophage-platelet aggregate formation and platelet accumulation in the subendothelium. Importantly, platelet CLEC-2 deficiency reduced plaque formation in atherosclerotic mice. Further mechanistic investigation may help elucidate how circulating platelets accumulate in the atherosclerotic plaques and the role of accumulated platelets in atherosclerosis.

In summary, we showed that platelets accumulate in the subendothelium and in the atherosclerotic plaques by d-flow in mice, which may play a pivotal role in the recruitment and transmigration of monocytes during the initiation of atherosclerosis. These data extend our knowledge of the early events in atherogenesis and unveil the importance of platelets for d-flow-induced vascular inflammation and atherosclerosis.

## Materials and Methods

### Mice

All animal procedures described in this study were performed using 8- to 16-week-old mice and were approved by the Institutional Animal Care and Use Committee of Soochow University (20140431). For platelet specific deletion of CLEC-2 expression, *Clec2^fl/fl^*; *Pf4-Cre* mice were created by crossing *Clec2^fl/fl^* mice with *Pf4-Cre* mice (stock number 008535; C57BL/6J and Sv129 background; Jackson Laboratory). For hematopoietic deletion of PDPN expression, *PDPN^fl/fl^*; *LysM-Cre* mice (Mye *Pdpn^-/-^*) were created by crossing *PDPN^fl/fl^* mice with *LysM-Cre* mice (stock number 004781; C57BL/6J and Sv129 background; Jackson Laboratory). *Selplg^-/-^* mice were obtained from the Jackson Laboratory (stock number 004201, C57BL/6J background).* ApoE^-/-^* mice (stock number T001782, C57BL/6J background), *Ldlr^-/-^* mice (stock number T001464, C57BL/6J background), C57BL/6J mice (stock number N000013) and EGFP (stock number NM-TG-00005, C57BL/6J background) mice were purchased from Shanghai Model Organisms Center. All mice were housed in a pathogen-free facility at an ambient temperature of 22 °C to 25 °C, humidity and light cycle (12:12 h light: dark), and free access to water and food. All mice were backcrossed on C57BL/6J or Sv129 for > 10 generations.

### Animal models

We performed partial carotid ligations (PCL) *in vivo* by ligating three branches of left carotid artery, leaving the superior thyroid artery untied. The intact right common carotid artery (RCA) served as a control as described previously 39. Briefly, the external carotid artery (ECA), internal carotid artery (ICA), and occipital artery (OA) of the left common carotid artery (LCA) were ligated, leaving the superior thyroid artery (STA) intact. Ultrasound Biomicroscopy (Vevo 2100, VisualSonics) was used to examine blood flow 24 h after the procedure. A reduction of blood flow by ~80% in the downstream of the ligation line (the distal 1/4 to 1/2 position) of LCA compared to the right carotid artery (RCA) and appearance of diastolic regurgitation indicated successful establishment of the PCL model. For atherosclerosis studies, the *ApoE^-/-^* or *Ldlr^-/-^* male mice on a normal chow diet were switched to a high-fat diet (HFD) (0.15% cholesterol and 21% fat without added cholate, Harlan Teklad, 88137, USA) for the next 4 weeks before the aortic arch sectioning or for the next 1, or 4 weeks after PCL before LCA sectioning.

### Whole-mount staining

*En face* whole-mount immunostaining was performed on the luminal side of common carotid arteries as described previously 39. In brief, dissected arteries were fixed in 4% paraformaldehyde (PFA) for 4 h and washed with 0.3% PBSTX (0.3% Triton X-100 in PBS) for 4 times (15 min/time) at room temperature (RT). After washing, samples were blocked with 3% PBSMT (3% non-fat milk in PBSTX) at 4 °C overnight and then incubated with a primary antibody (in 3% PBSMT) at 4 °C overnight. After washing with 0.3% PBSTX for 4 times (30 min/time), the samples were incubated with a fluorescent-conjugated secondary antibody (1 μg/ml in 0.3% PBSTX) at 4 °C overnight. After washing with 0.3% PBSTX for 4 times (30 min/time), the samples were incubated 15min with DAPI at room temperature, then washed 2 times and were fixed with 1% PFA for 3 min and washed with 0.3% PBSTX prior to mounting with cover slides and an anti-fading agent (0100-01, Southern Biotech, USA). Antibodies used in the *en face* immunostaining: rabbit anti-mouse CD31 (ab28364, Abcam, USA), sheep anti-mouse CD42d (AF6990, RD, USA), rat anti-mouse F4/80 (MAB5580, R&D, USA), Syrian Hamster anti-mouse podoplanin antibody (14-5381-82, eBioscience, USA).

### Immunofluorescence staining

Cross-sections of carotid arteries were used for confocal microscopic imaging. Platelets were stained with antibodies against CD42d (AF6990, R&D, USA), CD41 (553847, BD, USA) and PF4 (ab282111, Abcam, USA). Monocytes/macrophages were stained with rat anti-mouse F4/80 antibody (MAB5580, R&D, USA). Neutrophils were stained with Ly6G antibody (sc-52515, SANTA CRUZ, USA). Endothelial cells were stained with rabbit anti-mouse CD31 (ab28364, Abcam, USA). Podoplanin expression was determined with Syrian Hamster anti-mouse podoplanin antibody (14-5381-82, eBioscience, USA). Cytoskeletal proteins were determined with phalloidin antibody (CA1610, Solarbio, China). Secondary antibodies Alexa Fluor 488-conjugated donkey anti-rat IgG, Alexa Fluor 488-conjugated goat anti-mouse IgG, Alexa Fluor 647-conjugated donkey anti-rabbit IgG, and Alexa Fluor 647-conjugated goat anti-Syrian hamster IgG were all from Abcam. Donkey anti-sheep IgG NorthernLights™ NL557-conjugated antibody was purchased from R&D. Tissues were counterstained with DAPI before being mounted and examined by confocal microscopy, an Olympus multiphoton laser scanning microscope with high S/N ratio objectives and suppressed autofluorescence, which enables advanced deeper imaging with high resolution (FV1000MPE and FV3000MPE Olympus, Japan).

### Transmission electron microscopy

Five mm of carotid arteries of d-flow region were fixed in 2.5% glutaraldehyde at 4 °C overnight. The samples were washed twice with 0.075 M phosphatebuffered saline (PBS), fixed in 1% osmium tetraoxide (OsO4) for 60 minutes, and washed thrice. After dehydration with 30%, 50%, 70%, 80%, and 90% acetone for 15 minutes each and additional two final 100% acetone, samples were embedded in beam capsules. Ultrathin 60- to 90-nm-thick sections on grids were stained with uranyl acetate and observed under a transmission electron microscope (Tecnai G2 spirit BioTwin, USA).

### Generation of chimeric mice

Chimeric mice were generated as described previously 40. Briefly, *Clec2^fl/fl^* (WT)* and Clec2^fl/fl^; Pf4-Cre* (Plt *Clec2^-/-^*) donor male mice (8 w) were sacrificed and bone marrow cells were prepared. The recipient *Ldlr^-/-^* male mice (8 w) were fed with 80,000 U gentamicin/bottle of acidified water each week before and after the irradiation. The mice were irradiated with a dose of 8.5 Gray and transplanted with (5~10) ×10^6^ bone marrow cells from donor mice by tail vein. After bone marrow transplantation for 6 weeks, 5 µl of blood was collected from the retroorbital venous plexus, and CLEC-2 (146103, BioLegend, USA) expression on platelets (133914, BioLegend, USA) was analyzed by flow cytometry to determine the transplantation efficiency.

### Platelet isolation and infusion

Platelets were isolated as previously reported 40. In brief, whole blood was drawn from the inferior vena cava of mice into a syringe containing 1/6 volume of Acid-Citrate-Dextrose (39 mM citric acid, 75 mM sodium citrate, 135 mM dextrose; ACD). Blood samples were allowed to rest at room temperature for 5 min and centrifuged at 1900 g for 1 minute. Platelet-rich plasma (PRP) was obtained by centrifugation of blood at 800 rpm for 5 min. PRP was further centrifuged at 2800 rpm for 5 minutes to obtain platelet pellets. Platelets were washed twice and resuspended in Tyrode's buffer (137 mM NaCl, 2.8 mM KCl, 1 mM MgCl2, 12 mM NaHCO3, 0.4 mM Na2HPO4, 5.5 mM glucose, and 10 mM Hepes (pH 7.4) with 1 µM PGE1 and 0.02 U/ml Apyrase. Platelets were counted on an Automatic Hematology Analyzer (Sysmex, KX-21N) and adjusted to the desired concentration. For platelet transfusion, isolated platelets from EGFP mice (1×10^8^) were infused into the jugular vein of WT mice that have been subjected to PCL on LCA for 2 days. The infused EGFP^+^ platelets were allowed to circulate for 24 hours. LCAs and RCAs were then dissected for further analysis.

### Isolation of murine peritoneal macrophages

Isolation of murine peritoneal macrophages is as described previously 41. In brief, each C57BL/6J mouse was injected with 3 ml of 4% Brewer thioglycolate medium into the peritoneal cavity (T9032, Sigma, USA). After 72 hours, the mice were euthanized and sterilized with 75% alcohol. The induced macrophages were harvested and cultured with DMEM(H) for 4 hours at 37 °C. The non-adherent cells were discarded and the adherent macrophages were continued to culture in fresh medium for 12 hours. Macrophages were put into a cone-and-plate shear device for experimentation.

### Cell culture and the expression of podoplanin

Mouse monocyte/macrophage RAW264.7 cells and primary peritoneal macrophages of mice were cultured using DMEM(H) with 10% FBS, 100 U/ml penicillin and 0.1 mg/ml streptomycin. A modified cone-and-plate shear device was used to generate oscillatory shear (OS) and laminar shear (LS) *in vitro* 42. Cells (70% confluent) were stimulated with OS for 12 hours (10 ± 5 dyn/cm^2^) following pretreatment with LS for 12 hours (30 dyn/cm^2^). The adherent cells were measured for mRNA and protein expression. In mRNA assay, RAW264.7 cells and primary peritoneal macrophages were collected by RNAsimple Total RNA Kit (DP419, TIANGEN, China). For protein assay, RAW264.7 cells and primary peritoneal macrophages treated with or without OS were incubated by anti-PDPN antibody (127407, BioLegend, USA) at 37 °C for 15 minutes. After brief wash with PBS, 20000 cells per sample were examined by flow cytometry.

### Flow cytometry

For platelet-monocyte aggregate experiments, heparin anti-coagulated blood was collected from the abdominal aorta of mice and incubated with CD16/32 antibody (101320, BioLegend, USA) to block Fc receptors on the surface of immune cells to prevent subsequent non-specific staining. Blood was fixed with 4% PFA and then red blood cells were lysed to obtain leukocytes. Ly6C (128011, BioLegend, USA) and CD11b (101206, BioLegend, USA) double-positive cells were defined as monocytes, and CD41 antibodies (ab95725, Abcam, USA) were used to stain circulating platelets, and the platelet-monocyte aggregates (MPA) was shown as the percentage of total monocytes counted. For identification of *Plt Clec2^-/-^* mice, the expression of CLEC-2 on platelets was confirmed by CD41 (133914, BioLegend, USA) and CLEC-2 (146103, BioLegend, USA) antibodies, and the expression of CLEC-2 on myeloid cells was confirmed by CD11b and CLEC-2 antibodies. For identification of *Selplg^-/-^* mice, leukocytes were isolated and the expression of PSGL-1 on leukocytes was confirmed by CD45 (557235, BD, USA) and CD162 (51-1621-80, eBioscience, USA) antibodies.

### Migration and invasion assays

The 24-well transwell chambers (3422, Corning, US) were used for the cell invasion assay in accordance with the manufacturer's protocol. Briefly, 5×10^4^ RAW264.7 cells in 10% FBS DMEM(H) were added to the upper chamber and platelets were added to the compartment in a 100:1 ratio, and 500 µL of DMEM(H) containing 20% FBS was added to the lower chamber for culture. The cells were cultured in a humidified atmosphere for 10 or 20 hours at 37 °C in 5% CO2 and the non-invading cells were removed. The cells were then fixed with 4% PFA for 10 minutes and stained using 0.5% crystal violet (C0121, Beyotime, China) for 5 minutes, then washed 3 times in PBS. The migrating cells were counted under a phase-contrast microscope (DMIL LED, Leica, Germany). An alternative method to analyze the data was to cut out the membrane of the transwell chamber, fixed with 4% PFA, and stained with phalloidin and DAPI. The number of migrated cells can be observed by confocal microscopy.

### Inner layer (media and endothelium) separation

Dissolve 0.5 mg collagenase type II (0.5 mg/ml) in 1 ml HBSS to prepare 2× collagenase stock, sterilize through filtration. Incubate each carotid separately in 1 ml collagenase solution for 10 minutes at 37 °C. To stop collagenase reaction, add 1 ml HBSS containing 20% FCS. Peel the carotid adventitia with 2 microdissection forceps. The inner layer (Media and endothelium) of carotid were stored in liquid nitrogen for RNA extraction.

### Real-time quantitative PCR (RT-qPCR)

Total RNA was obtained from carotid artery by miRNeasy Mini Kit (NO.217004, QIAGEN, Germany) and reverse transcribed into cDNA using 5× All-In-One RT Mastermix (G490, Abm, China). RT-qPCR for specific genes was performed using SYBR Green PCR master mixture (Thermo Fisher) with custom-designed primers on the Roche LightCycler480 Real-Time PCR System. Results were normalized to GAPDH RNA, and the fold change was determined using the 2-ΔΔCT method. The following primers were used:

PDPN 5'GAGGCTCCAACGAGATCAAG-3' and 3'-CAGTAGCACCTGTGGTTGTT-5'.

### LPS-induced systemic inflammation

The mice were treated with 20 mg/kg LPS (L2880, Sigma, USA) or saline for one day, and heparin anticoagulated peripheral blood was collected. Number of white blood cells (WBC) were quantified using an automated hematology analyzer. The remaining blood was centrifuged (3000 rpm, 15 minutes) to obtain plasma samples. C-reactive protein (CRP) from plasma was detected by a biochemical analyzer and the presence of interleukin-1β (IL-1β) and TNF-α in plasma sample was determined by mouse IL-1β (1210122, Dakewe, China) and TNF-α ELISA Kit (1217202, Dakewe, China). The infiltration of platelets and monocytes on LCA was detected by* en face* staining.

### Image analysis

Whole-mount and cross-section staining were performed under an Olympus FV1000 or FV3000 confocal microscope with objective lens ranging from 20x to 40x, and spectral laser at wavelengths of 405, 488, 561 and 633 nm. Images of z-stacks with step size 0.3 µm or 2 µm were analyzed by Olympus FV3000 software. 3D images and rotating movies were generated in Imaris 3D/4D Visualization & Analysis software (Version 9.5, Bitplane AG, Zurich). Statistics about F4/80^+^ or CD42d^+^ fluorescence area was calculated by image J. The adjustments to brightness were applied equally across an entire z-projected image.

The acquisition of the z-stack of each sample follows a common principle, that is, the signal of infiltrating cells goes from the weakest to the strongest, and then disappears (cross-section) or the DAPI signal appears from endothelial cells or infiltrating inflammatory cells to smooth muscle cell layer nuclear signals (*en face*). Those z-stacks were then opened in Imaris imaging software. As shown in movie S1, the confocal 3D rendering was turned into a vascular surface. We calculated the area and volume of platelets in the field of view by constructing a surface. Then we used the Imaris surface contact area XTension to quantify the percent overlap between the platelets and monocytes/macrophages or neutrophils in 3D.

### Statistical analysis

All data were expressed as mean ± standard error of the mean and were compared by the nonparametric t-test for two groups by using GraphPad Prism software (San Diego, CA). P values < 0.05 were regarded as statistically significant. Normality of data was assessed via a Shapiro-Wilk normality test. Co-localization of platelets and P-selectin, and of PDPN and monocytes/macrophages were analyzed by cellSens 3.1 (Olympus, Japan).

## Supplementary Material

Supplementary figures and movie legends.

Supplementary movie 1.

Supplementary movie 2.

## Abbreviations

CLEC-2: C-type lectin-like receptor 2
CRP: C-reactive protein
D-flow: disturbed flow
ECA: external carotid artery
GPIbα: glycoprotein Ibα
HFD: high-fat diet
ICA: internal carotid artery
IL-1β: interleukin-1β
LCA: left carotid artery
MPA: monocyte-platelet aggregates
OA: occipital artery
OS: oscillating flow
PCL: partial carotid ligation
PDPN: podoplanin
RCA: right carotid artery
STA: superior thyroid artery
TEM: transmission electron microscopy
vWF: von Willebrand factor
WBC: white blood cells
3D: three dimensional
