# Supplementary material for: CLEC-2-dependent platelet subendothelial accumulation by flow disturbance contributes to atherogenesis in mice
Source: Theranostics. 2021 Oct 3;11(20):9791–804. doi: 10.7150/thno.64601 (PMC8581433; doi:10.7150/thno.64601)
Supplement: Supplementary file 1 — Supplementary figures and movie legends. [file thnov11p9791s1.pdf]

Supplemental Information

CLEC-2-dependent platelet subendothelial accumulation by flow disturbance contributes to atherogenesis in mice

Chaojun Tang[1,2,3*], Lei Wang[1*], Yulan Sheng[1], Zhong Zheng[1], Zhanli Xie[1], Fan Wu[1], Tao You[1], Lijie Ren[1], Lijun Xia[4], Changgeng Ruan[1,2], and Li Zhu[1,2,3]

[1]Cyrus Tang Hematology Center, Cyrus Tang Medical Institute, [2]Collaborative Innovation Center of Hematology of Jiangsu Province, [3]Suzhou Key Lab for Thrombosis and Vascular Biology, Soochow University, Suzhou, China.

[4]Cardiovascular Biology Research Program, Oklahoma Medical Research Foundation, Oklahoma City, OK, USA

[*] These authors contributed equally to this work.

Correspondence author: Li Zhu, Cyrus Tang Hematology Center, Soochow University, 215123, Jiangsu, China. Tel: 86-512-65880899; Fax: 86-0512-65880929; E-mail: zhul@suda.edu.cn

Supplementary Information

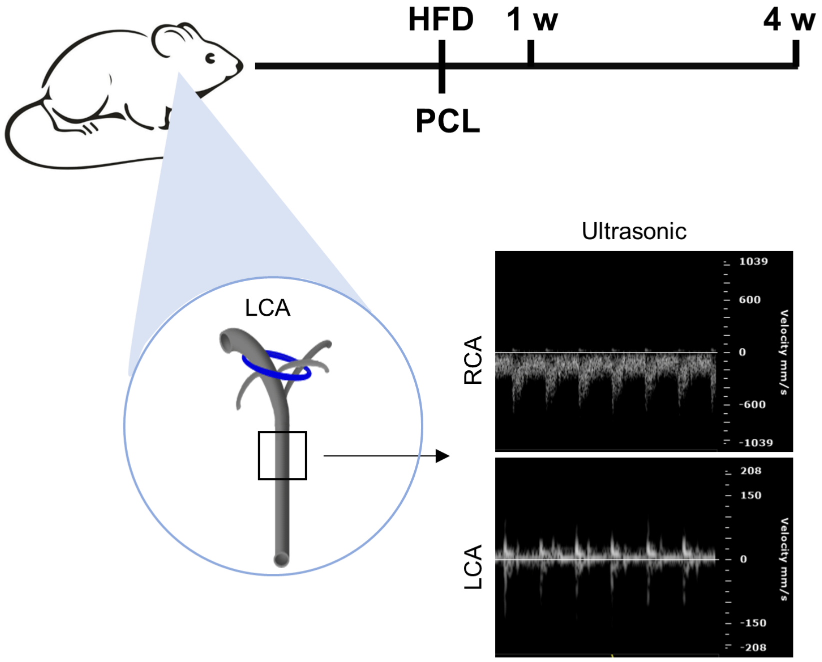

Figure S1. Construction of mouse carotid artery atherosclerotic plaque model induced by disturbed flow. Experimental overview: Adult *Ldlr*$^{-/-}$ mice or *ApoE*$^{-/-}$ mice were subjected to partial carotid ligation (PCL) surgery and fed with high-fat diet for 1 or 4 weeks to induce atherosclerotic plaque in the carotid artery. PCL surgery was performed by ligating the three branches of the left carotid artery (internal carotid artery, external carotid artery, and occipital artery), with only the superior thyroid artery left untouched so that the distal end of the left common carotid artery (LCA) generated disturbed flow (d-flow) (left panel). The blood flow direction and velocity of the distal 1/4~1/2 region of the mouse carotid artery were detected by ultrasonic system in the LCA PCL group and right common carotid artery (RCA) control group. The ligated left carotid artery showed slow blood flow velocity with the diastolic regurgitation, indicating the successful construction of the d-flow mouse model (right panel).

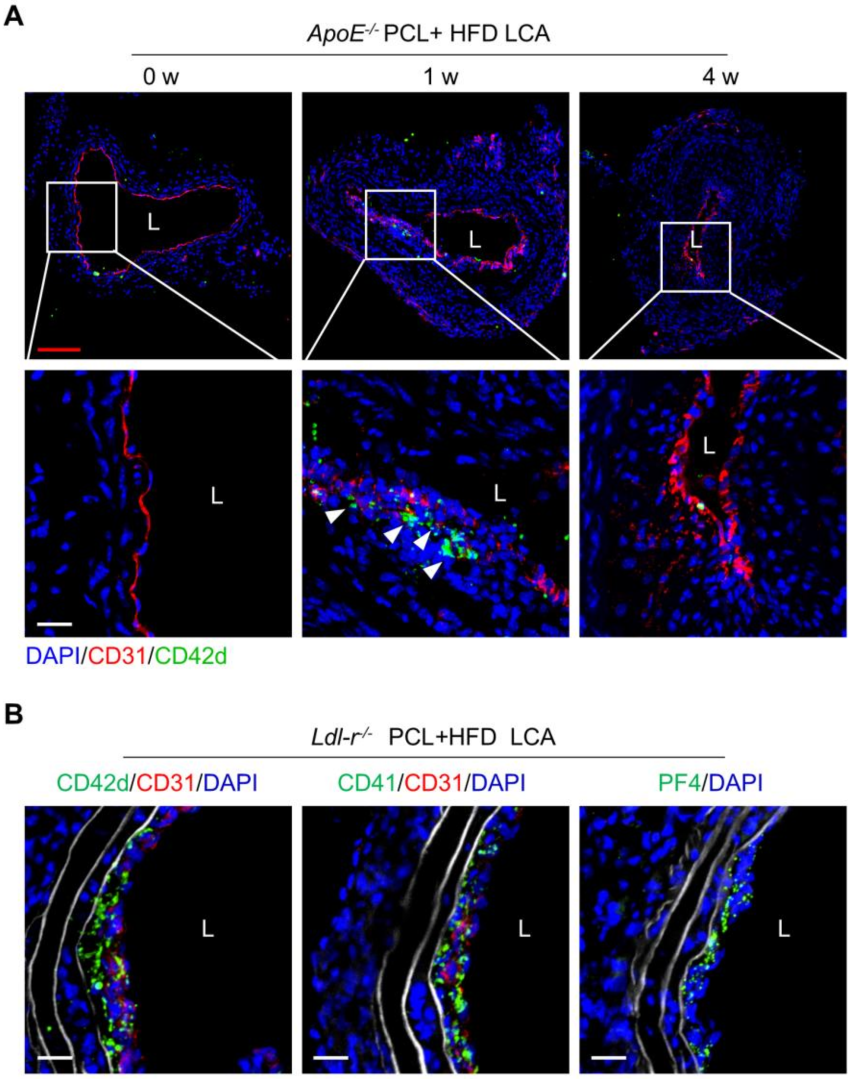

Figure S2. Platelets are detected inside atherosclerotic plaques. (A) The representative cross-section staining of carotid plaque from *ApoE*$^{-/-}$ mice on high-fat diet (HFD) for 1 or 4 weeks after PCL. Green, CD42d; blue, DAPI; red, CD31. L, lumen. White arrows indicate platelets. The bottom panel is the enlarged image of the box in the upper panel. Bar = 100 μm (upper) or 20 μm (bottom). (B) *Ldl-r*$^{-/-}$ mice were subjected to PCL surgery and then fed high fat diet for 1 weeks. The serial sections of the LCA were stained by immunofluorescence with antibodies against CD42d (left panel), CD41 (middle panels) and PF4 (right panel). Bar = 20 μm. L, lumen. Blue, DAPI; red, CD31; green, CD42d, CD41 or PF4; white, elastic fibers.

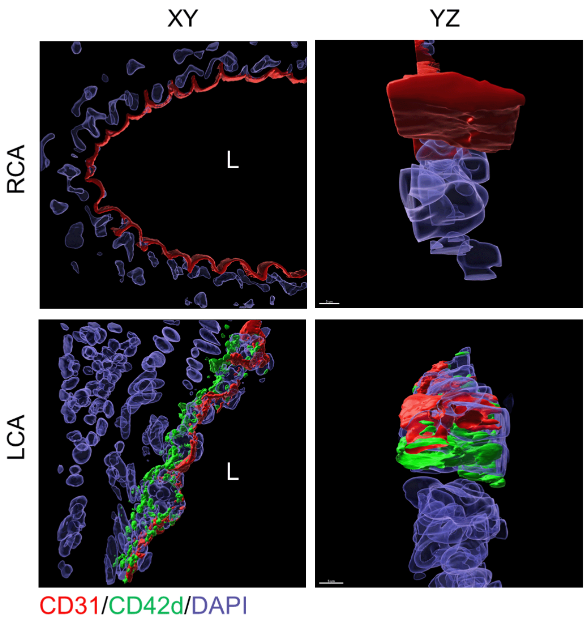

Figure S3. D-flow induces platelet subendothelial accumulation in mice. The representative 3D images of cross-section staining from RCA (upper) and LCA (bottom) after PCL for 2 days. The enlarged pictures in right panel are the original images in the left panel that were rotated for 90 degrees. Green, CD42d; red, CD31; blue, DAPI. L, lumen.

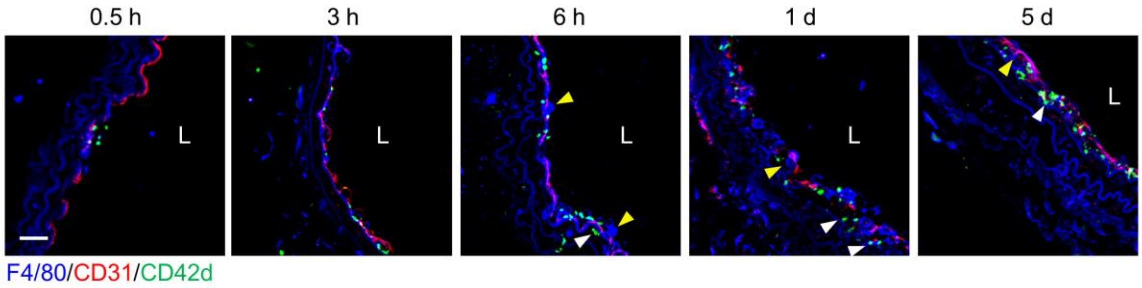

Figure S4. Platelet and monocyte/macrophage subendothelial accumulation by d-flow. The representative images of cross-section staining from LCA of PCL at different time points (0.5 h, 3 h, 6 h, 1 d, 5 d) after PCL for monocyte and platelet accumulation. L, lumen. Green, CD42d; red, CD31; blue, F4/80 and elastic fibers. White arrows indicate platelets and yellow arrows indicate monocytes/macrophages.

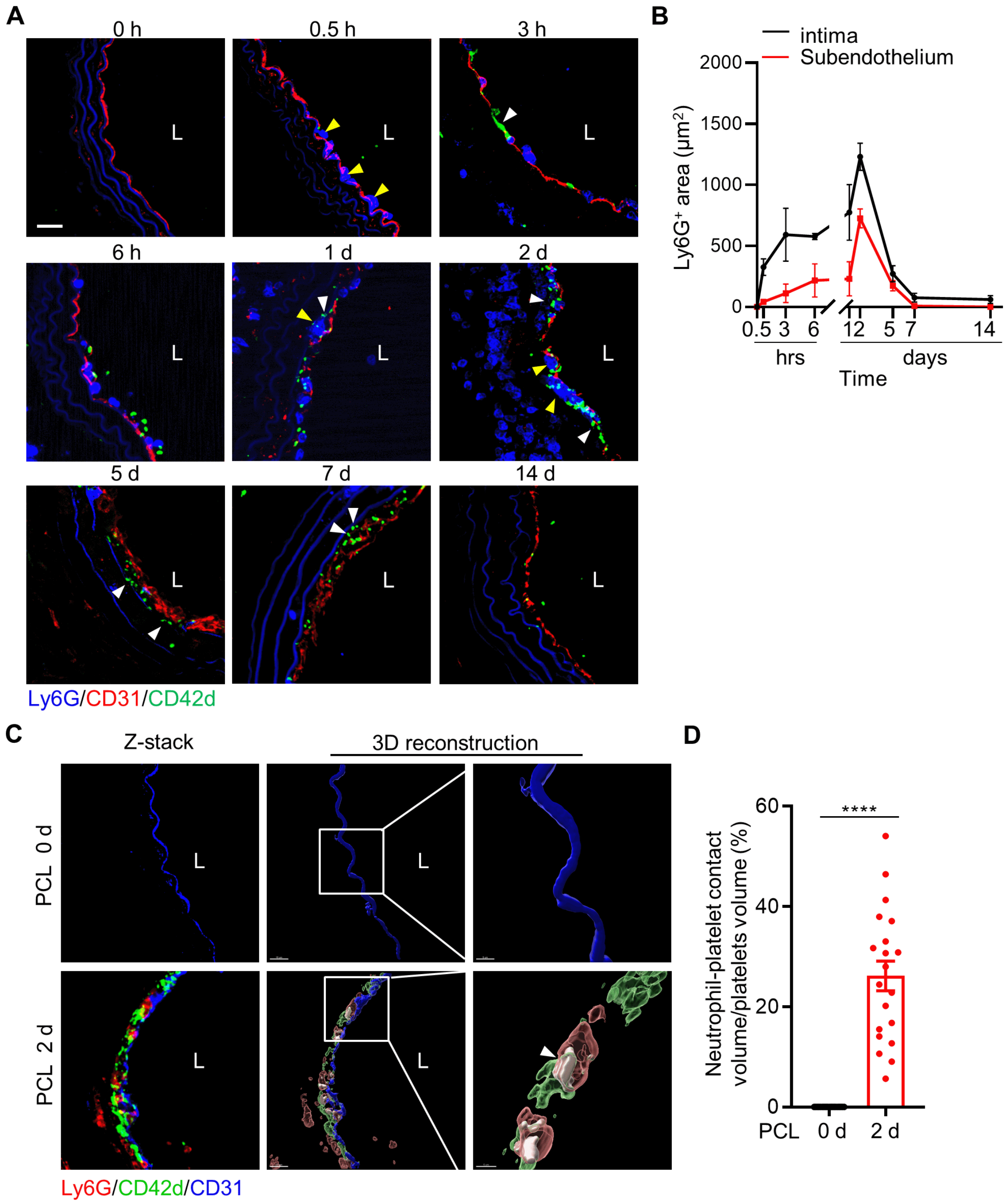

Figure S5. Platelet and neutrophil subendothelial accumulation by d-flow. (A) The representative images of cross-section staining from LCA of PCL at different time points (0 h, 0.5 h, 3 h, 6 h, 1 d, 2 d, 5 d, 7 d, 14 d) after PCL for neutrophil and platelet accumulation. L, lumen. Green, CD42d; red, CD31; blue, Ly6G and elastic fibers. White arrows indicate platelets and yellow arrows indicate neutrophils. (B) The curves represent the infiltration of neutrophil in intima (black line) and subendothelial (red line) at different time points after PCL. N ≥ 4 mice per group. (C) The representative images of cross-section staining and 3D

reconstruction image from LCA after PCL for 0 or 2 days to observe platelet-neutrophil aggregates. The enlarged images of the boxed area in the middle panel were shown on the right. L, lumen. Green, CD42d; red, Ly6G; blue, CD31; white arrows point to the contact area of platelets-neutrophils. Bar = 20 µm. (D) The quantitative statistics of the contact volume ratio of platelets with neutrophils in blood vessels after PCL for 0 or 2 days by Imaris 9.5. N = 20 fields from 3 mice per group. Data are mean ± SEM. ****$P < 0.0001$, by unpaired t-test.

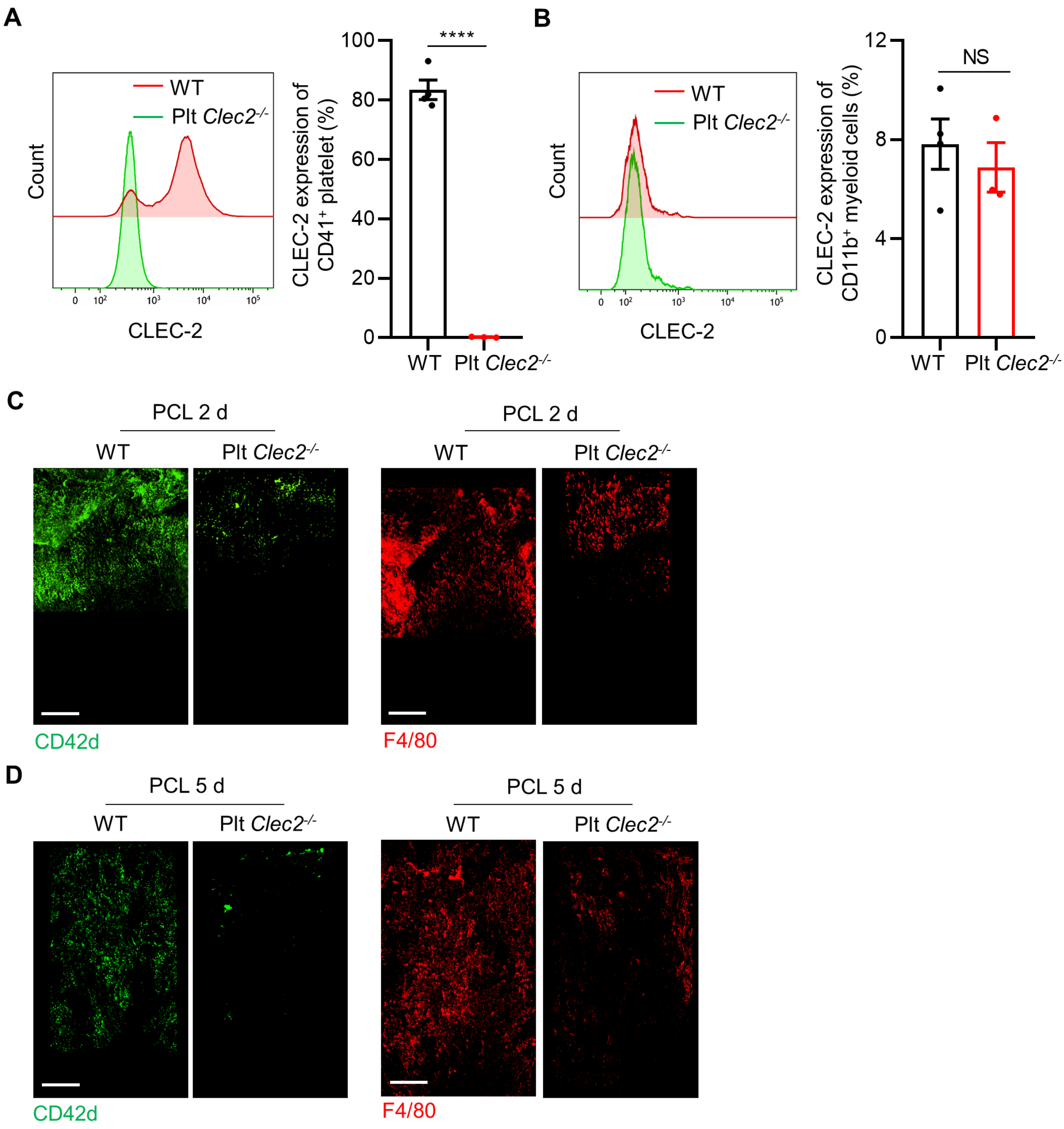

Figure S6. Loss of platelet CLEC-2 reduces monocyte and platelet interaction. (A, B) Flow cytometry analysis indicates that CLEC-2 in platelets, but not myeloid cells, was deleted in Plt *Clec-2*$^{-/-}$ mice. N = 3 mice per group. (C, D) The representative images of *en face* staining of platelet (left) or monocyte/macrophage (right) accumulation in the endothelium from WT or Plt *Clec2*$^{-/-}$ mice 2 (C) or 5 (D) days after PCL. Bar = 200 µm. Green, CD42d; red, F4/80.

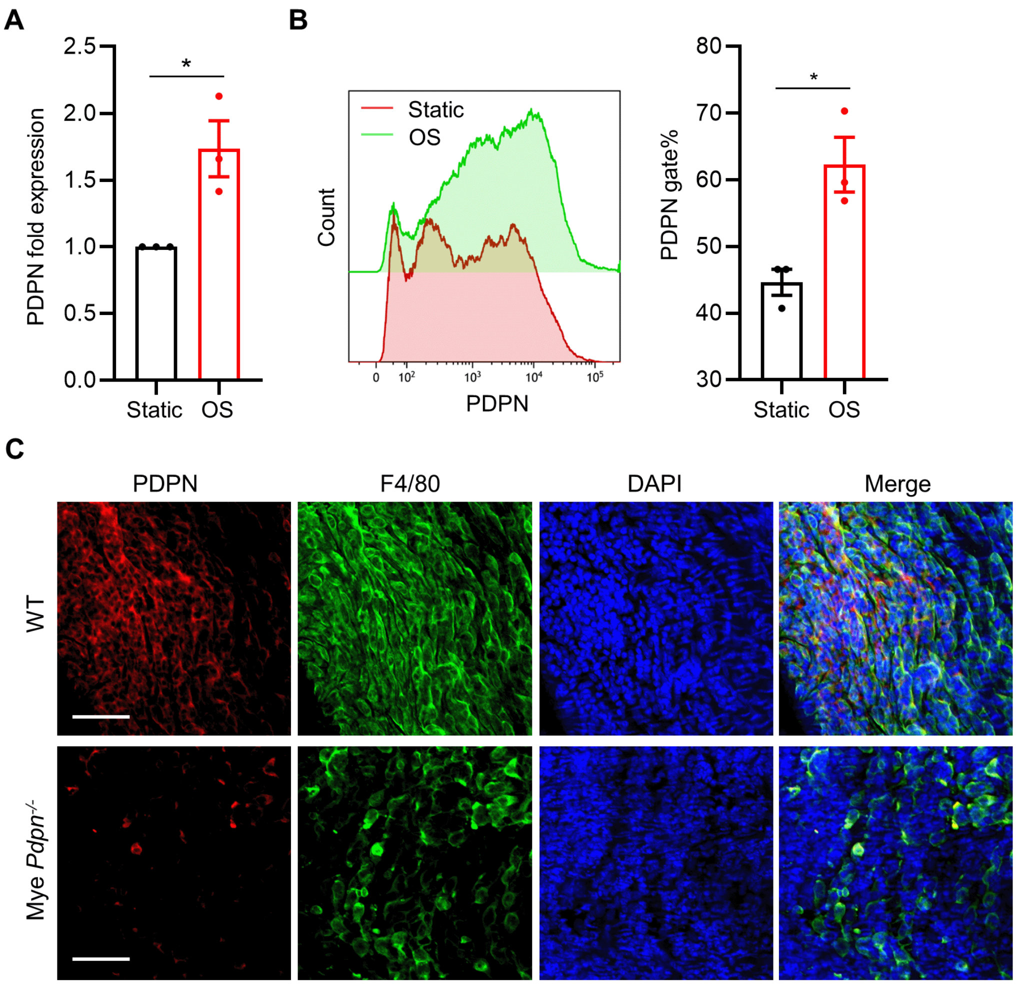

Figure S7. Disturbed flow promoted the expression of PDPN on primary mouse peritoneal macrophages. Peritoneal macrophages were isolated after intraperitoneal injection of 3 mL of a 4% thioglycolate broth for 72 h in C57BL/6J mice. Primary mouse peritoneal macrophages were treated for 12 h by oscillatory shear stress (OS) following laminar shear stress (LS) treatment for 12 h. The expression of PDPN was examined by qPCR (A) and flow cytometry (B). In (B), a representative histogram was shown in the left. N = 3 per group. Data are mean ± SEM. **P* < 0.05, by unpaired t-test. (C) Verification of myeloid *Pdpn*$^{-/-}$ mice. *Pdpn*$^{fl/fl}$ (WT), and *Pdpn*$^{fl/fl}$; *LysM-cre* (Mye *Pdpn*$^{-/-}$) mice were treated with PCL for 3 days, and *en face* staining of LCA was used to observe the expression of PDPN in infiltrating monocytes. Red, PDPN; green, F4/80; blue, DAPI. Bar = 50 μm.

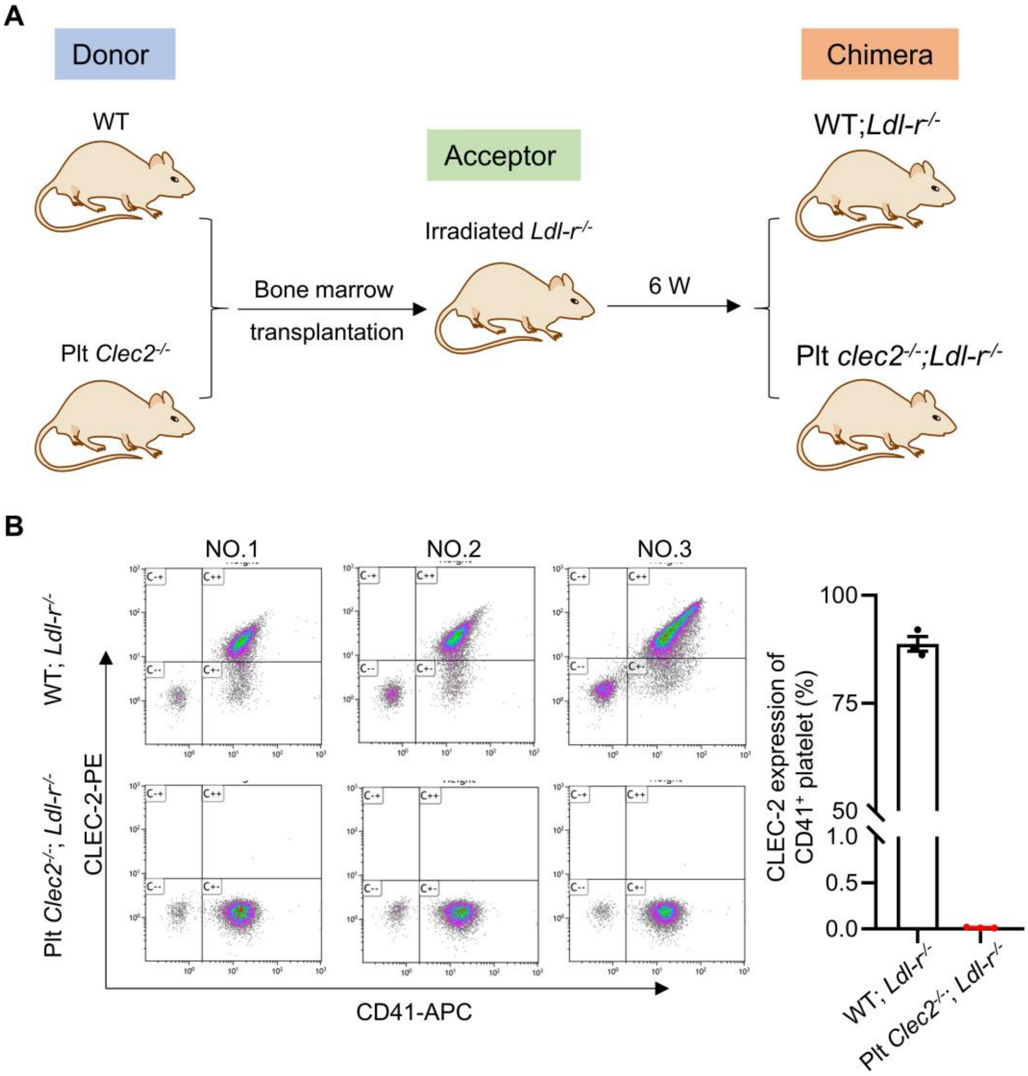

Figure S8. Experimental procedure and verification of bone marrow transplantation. (A) Experimental overview: bone marrow cells from WT or Plt *Clec2*$^{-/-}$ mice were injected into irradiated *Ldl-r*$^{-/-}$ mice through the tail vein. After 6 weeks, whole blood was collected for flow cytometric analysis of CLEC-2 expression in platelets. (B) Left: the representative flow cytometry analysis chart, the expression of CLEC-2 on platelets is represented by CD41 and CLEC-2 double positive. Right: the quantitative analysis of CLEC-2 expression on platelets in WT; *Ldl-r*$^{-/-}$ or Plt *Clec2*$^{-/-}$; *Ldl-r*$^{-/-}$ mice. The CLEC-2 expression on platelets is almost 0, indicating that the Plt *Clec2*$^{-/-}$ bone marrow chimeric *Ldl-r*$^{-/-}$ mice model was successfully constructed.

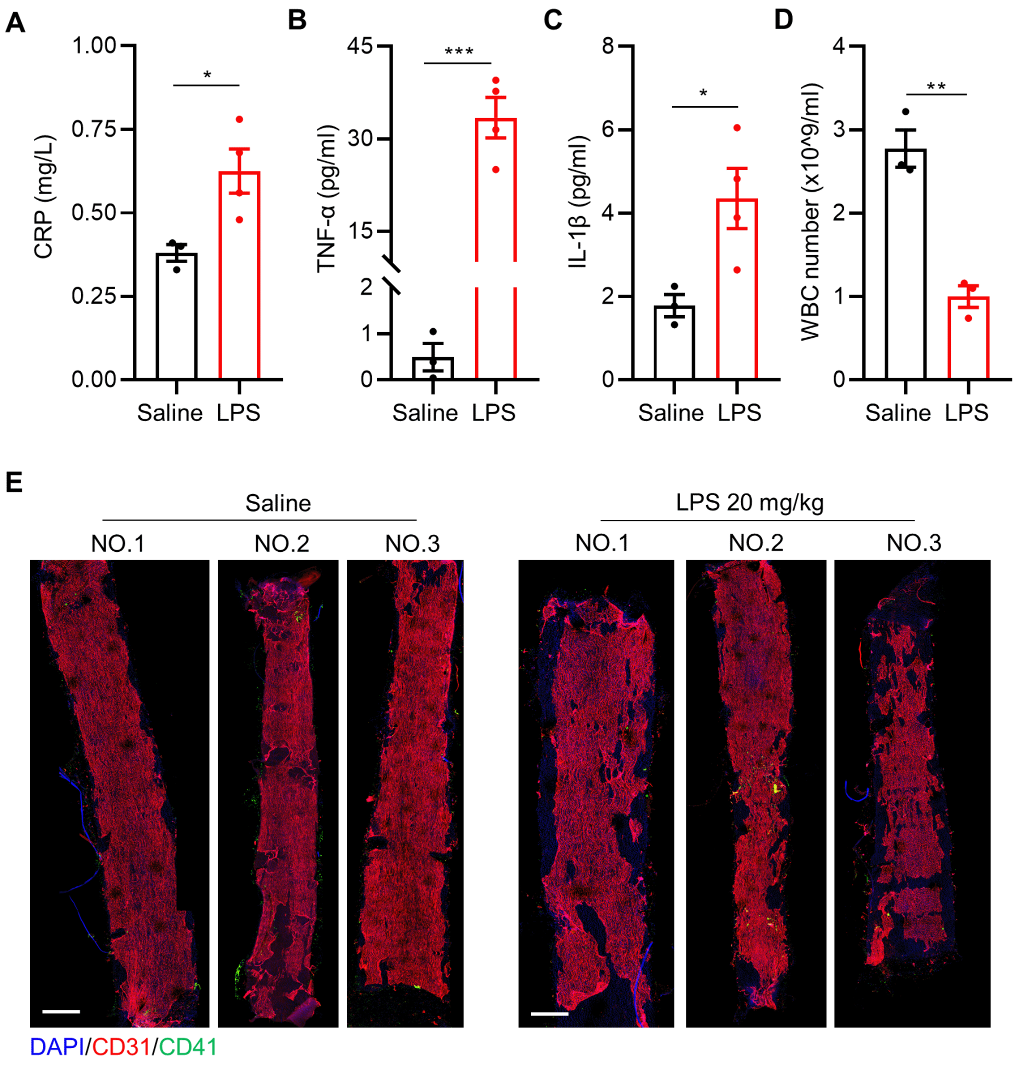

Figure S9. Platelet accumulation was not observed in LPS-induced vascular inflammation in mice. The mice were treated with 20 mg/kg LPS or saline for one day and the indicator for successful model construction is tested (A-D). (A) Increased plasma C-reactive protein (CRP) concentration in mice after LPS treatment. (B, C) Mouse TNF-α and IL-1β ELISA assay from plasma. (D) Number of white blood cells (WBC) were quantified using an automated hematology analyzer. N ≥ 3 mice per group. Data are mean ± SEM. **P* < 0.05, ***P* < 0.01, ****P* < 0.001, by unpaired t-test. (E) The platelets on the carotid artery were examined by *en face* staining. Green, CD41; red, CD31; blue, DAPI. Bar = 500 μm.

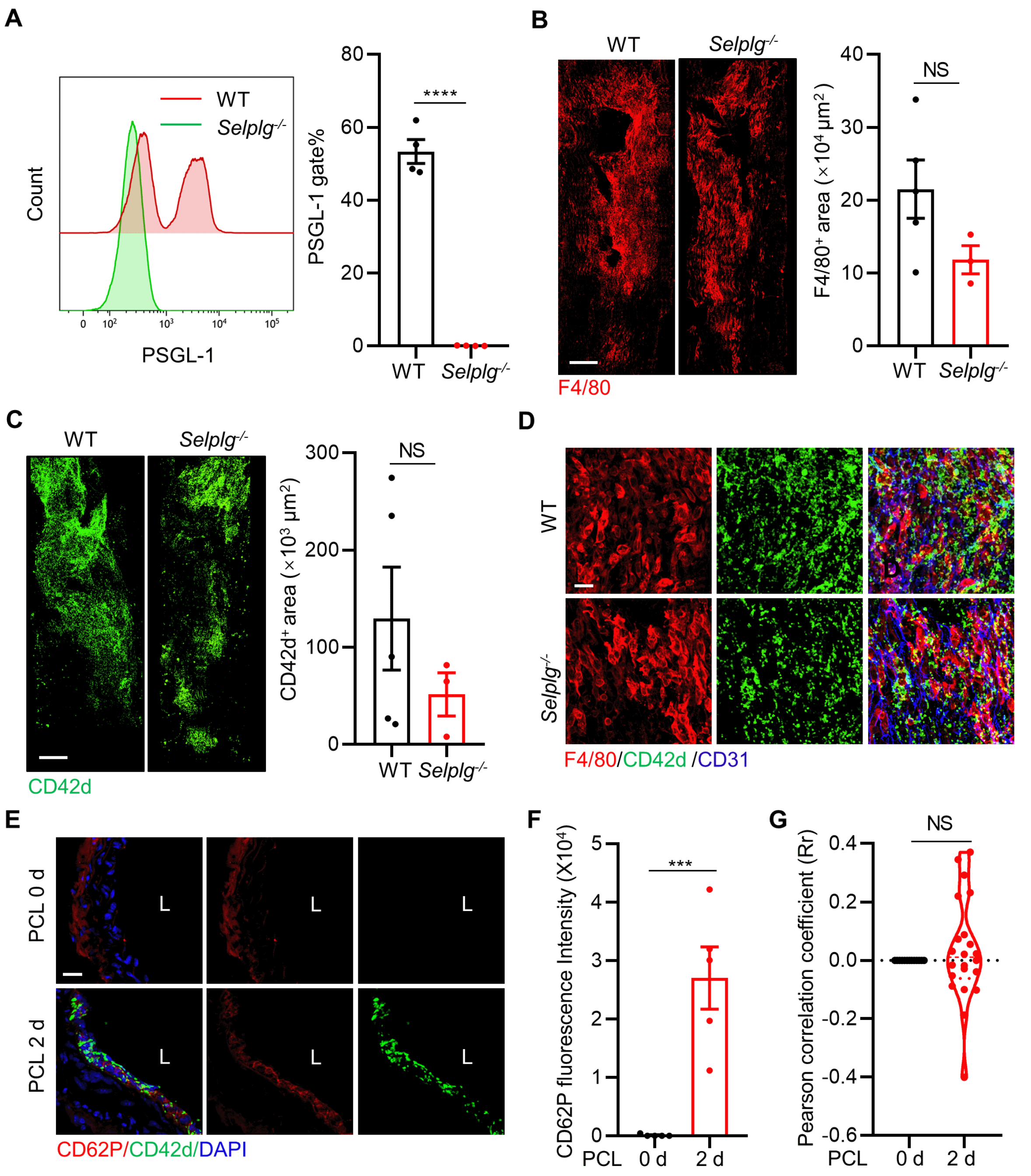

Figure S10. PSGL-1 deletion did not affect the subendothelial accumulation of platelets and monocytes/macrophages after PCL 2 days. (A) PSGL-1 deletion in leukocytes of *Selplg*$^{-/-}$ mice was validated by flow cytometry. N = 4 mice per group. (B) Left: the representative images of *en face* staining of monocyte/macrophage accumulation in the endothelium from WT or *Selplg*$^{-/-}$ mice 2 days after PCL. Bar = 200 μm. Red, F4/80. Right: the quantitative analysis of F4/80^{+} monocyte/macrophage area in the distal of LCA (where d-flow usually occurs in PCL). N ≥ 3 mice per group. (C) Left: the representative images of

en face staining of platelets accumulation in the endothelium from WT or *Selplg*$^{-/-}$ mice 2 days after PCL. Bar = 200 μm. Green, CD42d. Right: the quantitative analysis of CD42d^{+} platelet area. N ≥ 3 mice per group. (D) The representative *en face* staining images of the infiltrated region of LCA from WT or *Selplg*$^{-/-}$ mice 2 days after PCL. Red, F4/80; green, CD42d; blue, CD31. Bar = 20 μm. (E) The representative images of cross-section staining of platelet and P-selectin from LCA after PCL 2 days. Green, CD42d; red, CD62P; blue, DAPI; Bar = 20 μm. (F) Quantitative analysis of CD62P positive fluorescence intensity on inner elastic fiber from WT mice 2 days after PCL. N ≥ 5 fields per group. (G) Co-localization analysis of platelets and P-selectin by cellSens 3.1. The ordinate represents Pearson correlation coefficient (PCC; Rr), the abscissa represents the group. N ≥ 22 fields per group. In all relevant panels, data are mean ± SEM. NS*P* > 0.05, ****P* < 0.001, *****P* < 0.0001, by unpaired t-test.

Movie S1. 3D reconstruction of LCA from WT mice 2 days after PCL

Movie S2. Monocyte/macrophage-platelet aggregates and neutrophil-platelet aggregates in the intima 2 days after PCL
